# Aggregation-Induced Emission Poly(meth)acrylates for Photopatterning *via* Wavelength-Dependent Visible-Light-Regulated Controlled Radical Polymerization in Batch and Flow Conditions

**DOI:** 10.1021/acs.macromol.2c01413

**Published:** 2022-11-11

**Authors:** Congkai Ma, Ting Han, Spyridon Efstathiou, Arkadios Marathianos, Hannes A. Houck, David M. Haddleton

**Affiliations:** † Department of Chemistry, University of Warwick, Coventry CV4 7AL, United Kingdom; ‡ Center for AIE Research, College of Materials Science and Engineering, Shenzhen University, Shenzhen 518060, China

## Abstract

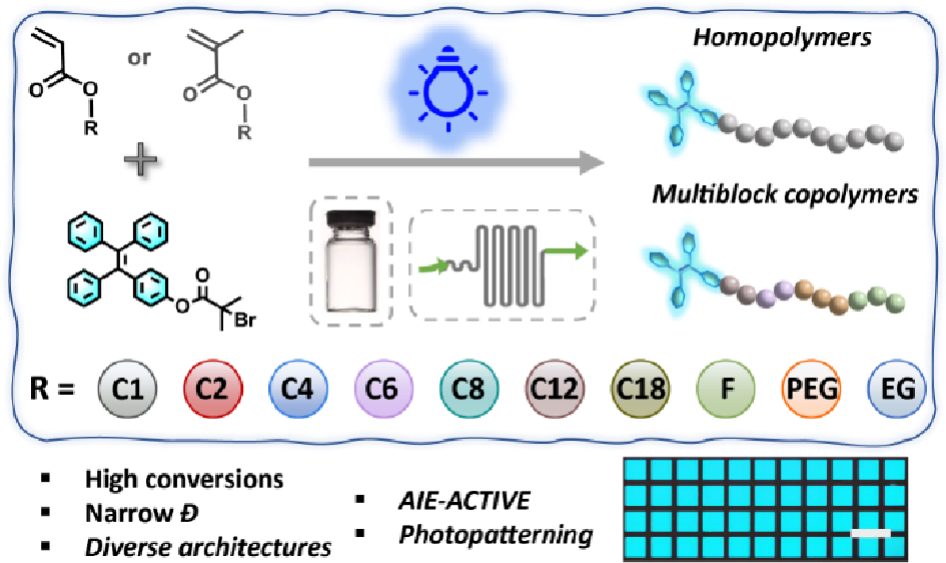

A robust wavelength-dependent visible-light-regulated reversible-deactivation radical polymerization protocol is first reported for the batch preparation of >20 aggregation-induced emission (AIE)-active polyacrylates and polymethacrylates. The resulting polymers possess narrow molar mass distributions (*Đ* ≈ 1.09–1.25) and high end-group fidelity at high monomer conversions (mostly >95%). This demonstrated control provides facile access to the *in situ* generation of complex sequence-defined tetrablock copolymers in one reactor, even while chain extending from less reactive monomers. Polymerizations can be successfully carried out under various irradiation conditions, including using UV, blue, green, and red LED light with more disperse polymers obtained at the longer, less energetic, wavelengths. We observe a red shift and wavelength dependence for the most efficient polymerization using LED illumination in a polymerization reaction. We find that the absorption of the copper(II) complex is not a reliable guide to reaction conditions. Moreover, the reported protocol is readily translated to a flow setup. The prepared AIE-active polymers are demonstrated to exhibit good photopatterning, making them promising materials for applications in advanced optoelectronic devices.

## Introduction

Luminescent materials have fundamental and technological significance, attracting both academic and commercial interest. Until the aggregation-induced emission (AIE) concept was coined, much effort was put toward applying aggregation-caused quenching effects of conventional luminophores with large conjugated coplanar molecular configurations.^1^ Comparatively, AIE-active molecules often possess twisted propeller-shaped structures, resulting in hindered intramolecular motion when aggregated. Accordingly, the emission of an AIEgen can be turned on in an aggregated state.^2^

An increasing array of AIE-containing polymeric systems have been explored *via* various synthetic methodologies, such as ring opening polymerization,^3^ polycoupling,^4^ click polymerization,^5−7^ and multicomponent polymerization.^8−10^ The resulting AIE-active polymers can exhibit excellent processability, stability, and biocompatibility, allowing for practical applications in diverse areas including optoelectronics, sensing, imaging, and biological therapy.^11−13^ Nevertheless, there is limited work concerning a facile synthesis of well-defined and narrow-disperse AIE polymers. Note that dispersity can be a key factor in polymer design and can impact material properties, for example, in self-assembly and mechanical performance.^14,15^ Controlled dispersity is often reported to be beneficial for systematic investigation and prediction of the structure–property relationships of macromolecules.^16−18^

Reversible-deactivation radical polymerization (RDRP), or controlled/living radical polymerization, including nitroxide mediated polymerization (NMP), reversible addition-fragmentation chain transfer (RAFT), and atom transfer radical polymerization (ATRP) polymerization, has revolutionized polymer science, enabling good control over diverse molecular topology, molecular weight, and chain length distributions.^16,19,20^ Other outstanding benefits of RDRP include the ability to perform chain extension with high tolerance to varying reaction conditions^21,22^ and chemical functionalities,^23−25^ collectively contributing to its robustness and versatility with respect to many vinyl monomers.^16,19,26−29^ In particular, a broad interest in light-mediated RDRP arises as it allows for spatial and temporal control over reaction kinetics, monomer sequences, and compositions *via* external regulation of the reversible activation–deactivation equilibrium using a renewable source of energy.^30−36^ Photochemistry can also offer sustainable reaction conditions in a move to supply energy by either photons or electrons in chemical synthesis. As such, functional materials are accessible employing RDRP with applications including nanotechnology^37,38^ and therapeutics.^20,39^

There are a few examples describing the synthesis of AIE polymers *via* RDRP via thermal and non-photochemical processes. Tang *et al.*(40) synthesized and screened a group of tetraphenylethene (TPE)-containing dithiocarbamates as RAFT agents and implemented them for the synthesis of AIE polymers with a range of functionalities. Furthermore, the fluorescence was exploited to visualize the polymerization process in real time. Bao *et al.*(41) prepared a library of polystyrenes with varied molar mass initiated by a bifunctional naphthalene diimide (NDI)-based AIE-active ATRP initiator. Note that benefiting from the excellent controllability of ATRP, the emission of the resulting polymers in the solid state could be finely tuned by precisely manipulating the monomer substituent variations, end-group transformation, and polymer chain length. There are still opportunities for the expansion of RDRP, in particular light-mediated protocols, for the preparation of diverse AIE polymeric materials with narrow chain length distributions, controlled chain length, and well-defined structures.^42^

Due to the differing solubilities of monomers and polymers and the different stabilities of radicals derived from monomers (e.g., acrylic, methacrylic, and styrenic), there is no one set of standard conditions for copper-mediated RDRP, or ATRP. For example, Cu(0) wire-induced RDRP is usually carried out in quite polar aprotic solvents such as DMSO, IPA, etc., which is very suitable for monomers and polymers soluble in these polar solvents and it is noted that these solvents also promote disproportionation of Cu(I) in the presence of aliphatic tertiary amine ligands such as Me_6_TREN and PMDETA. Polymerization in water of water-soluble monomers to water-soluble polymers is efficiently carried out, allowing Cu(I) to fully and rapidly disproportionate to Cu(0) and Cu(II) prior to addition of monomers and initiators. These aqueous conditions are very suitable for many acrylamides and water-soluble acrylates, which have high rates of polymerization. In our previous work, we have exploited the AIEgen-containing initiator tetraphenylethene bromoisobutyrate (TPEBIB) to generate a range of TPE-terminated polyacrylates and polyacrylamides with AIE properties *via* both Cu(0) wire-mediated and aqueous RDRP, respectively.^43,44^ AIE-terminated polymers have been reported widely as fluorescence probes for pH,^45^ temperature,^46^ and in bio-related areas.^47^ Among them, TPE-terminated poly(meth)acrylates were reported to have good film-forming ability and thermal stabilities, which could be used as potential fluorescence sensors for explosive detections,^48−50^ solution viscosity modifiers,^51^ and cell imaging.^43^

Effective controlled RDRP can also be carried out *via* the photoreduction of Cu(II) in the presence of a greater than two-fold excess of appropriate tertiary amine ligands, such as Me_6_TREN.^52^ In our previous work, we had hypothesized that irradiation occurs into both a free ligand absorbance and the alkyl bromide initiator and not into the copper(II) complex.^53,54^ Recently, Barner-Kowollik *et al*. probed the wavelength dependence of the photochemically induced copper-mediated polymerization of methyl acrylate between 305 and 550 nm, reporting the reactivities and comparing monomer conversion, molecular weights, and dispersity with the absorption spectrum of the copper(II) complex.^55^ For this, a specialized wavelength-tunable nanosecond pulsed laser polymerization (PLP) method was used to produce the so-called action plots at a constant photon flux while varying the excitation wavelength. Both the molecular weight and molecular weight distribution showed a wavelength dependence, illustrating that a choice over wavelength is desirable. This later work also reports that the absorption spectrum of the copper(II) complex does not represent a robust guide for monomer-to-polymer formation, illustrated with an observed red shift away from the absorbance maxima to give the most effective polymerization.

**Scheme 1:**
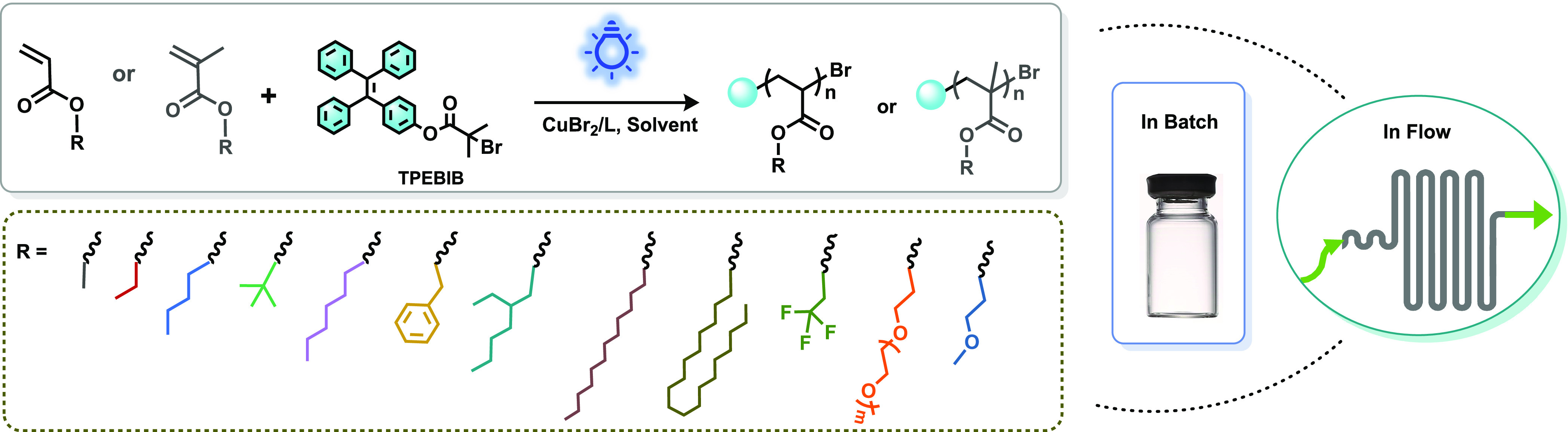
Reaction Scheme for the Polymerization of a Library of (Meth)acrylates with the Initiator TPEBIB *via* Visible-Light-Mediated Cu-RDRP in Both Batch and Flow Reactors

Herein, we combined our interest in the design of AIE-functional polymer materials with our continued investigations into light-induced RDRP by presenting the synthesis of a library of AIE-active polymers using visible-light-regulated RDRP in both batch and flow reactors (Scheme 1). Specifically, a total of 22 different monomers with diverse architectures were polymerized using a 1,1,2,2-tetraphenylethene functional ethyl α-bromoisobutyrate (TPEBIB) initiator. We further aimed to investigate the wavelength dependence of the TPEBIB-initiated polymerization of methyl acrylate in terms of both control and rate. For this, we used commercially available LED arrays in batch instead of relatively expensive, and generally inaccessible, PLP systems, thereby making it more transferable to many synthetic laboratories. We were also interested in how normal batch-type reactions would translate into a flow system using similar photochemical stimulation. This also allowed us to move away from quite hydrophilic polymers of our previous studies to many more relatively hydrophobic monomers/polymers, which form the majority of this type of polyacrylate and polymethacrylate used in bulk and film applications.

## Results and Discussion

**Figure 1:**
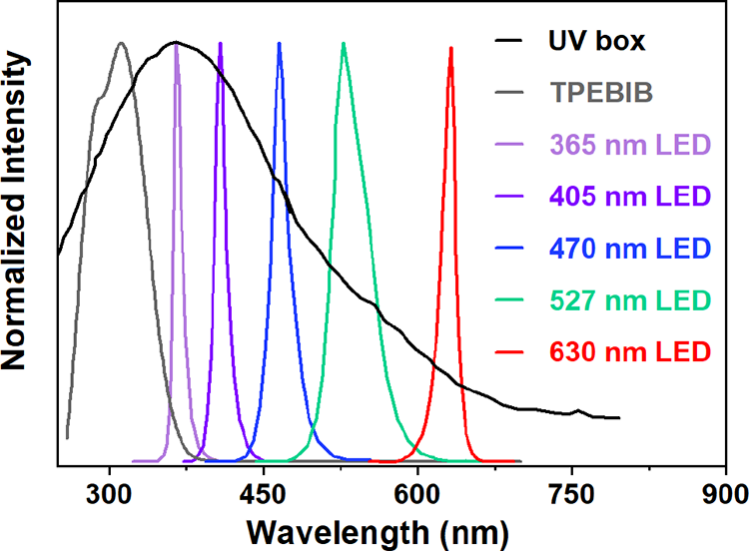
Normalized UV–Vis absorption spectrum of the initiator TPEBIB (DMSO) and spectral emission of the UV curing box (λ_max_ ≈ 360 nm) and LEDs used in this study.

**Figure 2:**
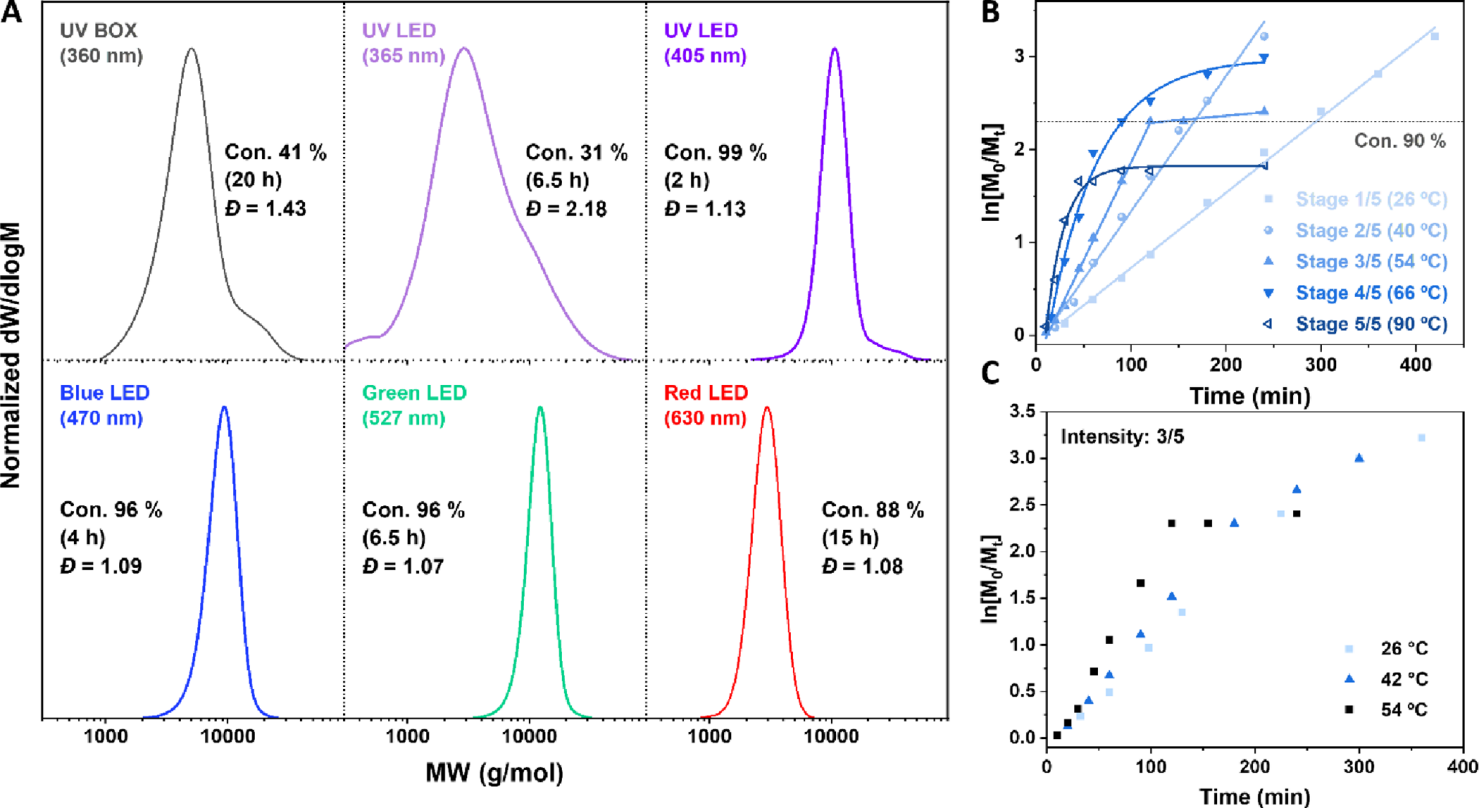
(A) THF-SEC-derived molecular weight distributions of TPE-PMA_100_ using the UV curing box and LED illumination with varying wavelengths. (B) Kinetics plots showing the effect of changing the blue (470 nm) LED intensity. (C) Effect of temperature to polymerization kinetics under the same blue light intensity (stage 3/5).

**Figure 3:**
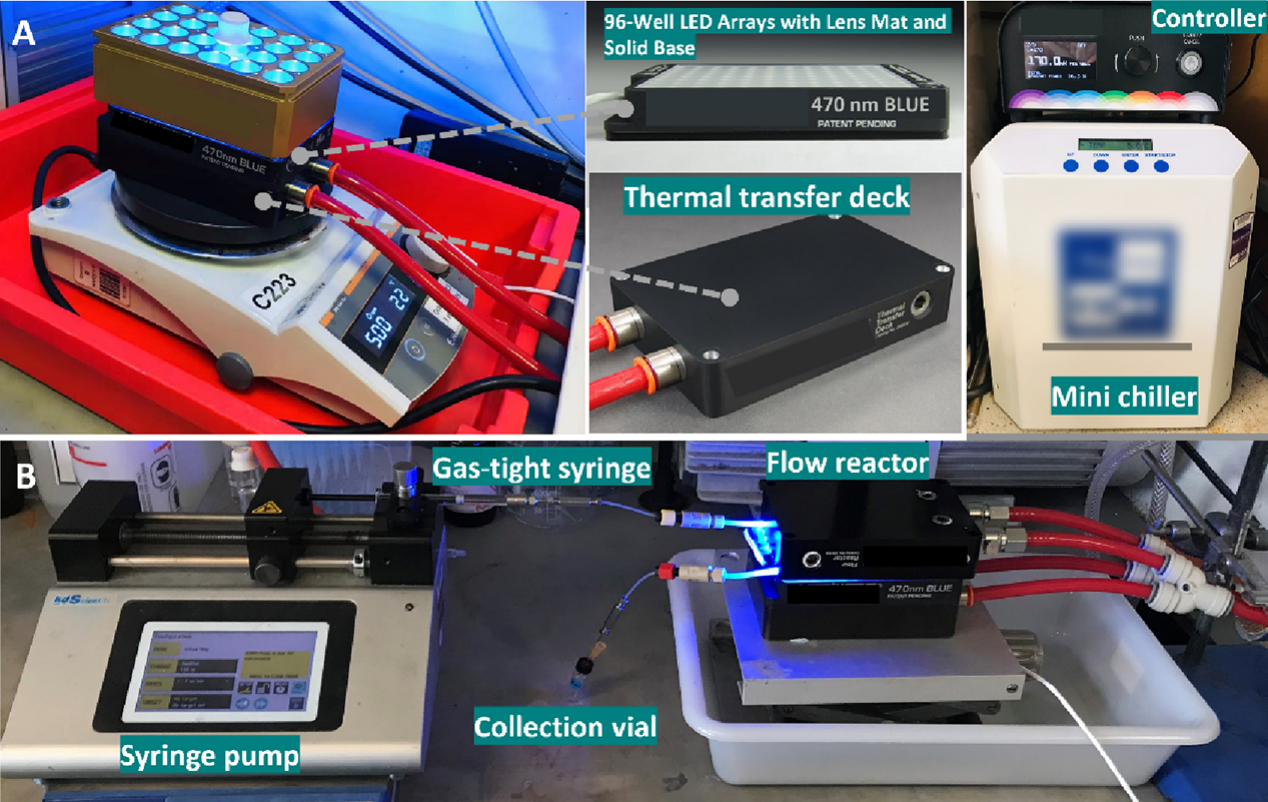
Reaction setup for photoreactions using a Lumidox II 96-Position LED Array in (A) batch and (B) flow reactors.

**Figure 4:**
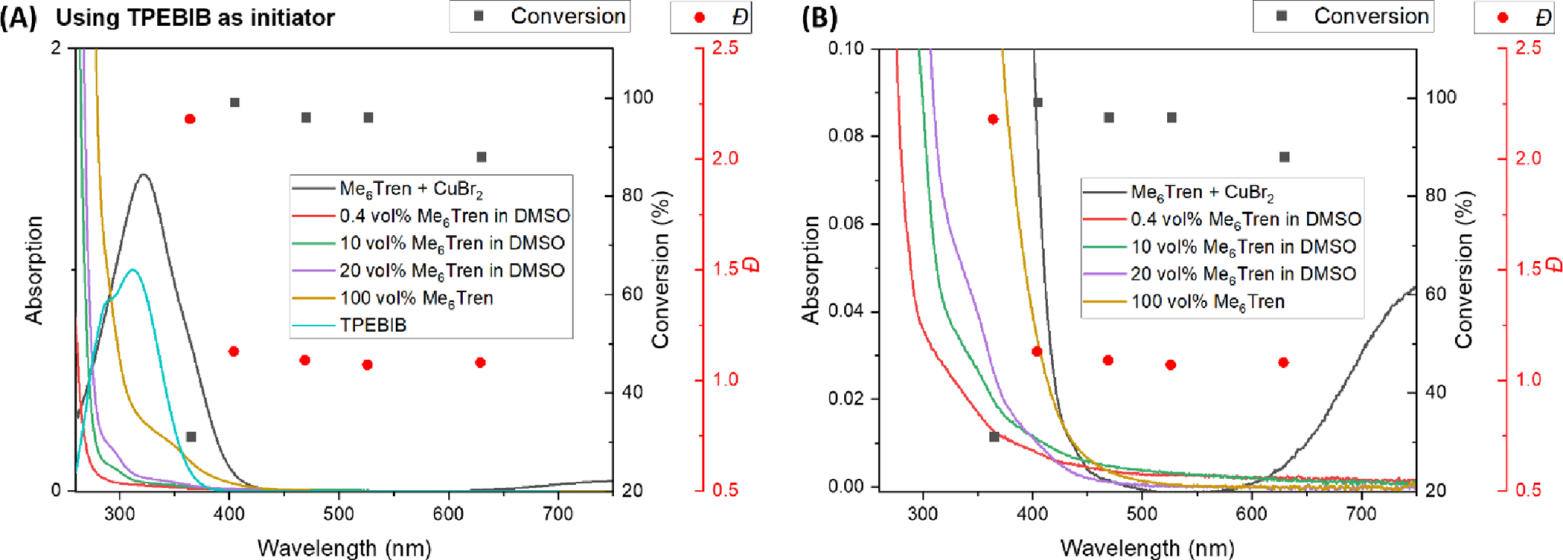
(A) Wavelength-dependent polymerization of methyl acrylate obtained by LED irradiation with overlaid UV–Vis spectrum of neat and diluted Me_6_TREN, CuBr_2_/Me_6_TREN in DMSO with reaction concentration, and the TPEBIB initiator in DMSO. Black points (squares) showing % conversion and red points (circles) showing the polymer dispersity. (B) Expansion of the absorption scale showing the spectral overlap of the Cu(II) complex with all light sources used.

Initially, the photoinduced homopolymerization of methyl acrylate (MA) (targeted DP_n_ = 100) was attempted using a standard method developed by our group^54^ using a UV curing box (broad band with λ_max_ ≈ 360 nm, Figure 1) with the UV emission ranging from <200 nm to well into the visible region. A sample after 20 h showed 41% monomer conversion by ^1^H NMR with the polymer having a dispersity of 1.43 (Figure 2A). A 96-well LED reactor, which is available from a commercial supplier with a wide range of single wavelengths generated from LEDs (Figure 1) in a simple interchangeable way, was also employed for a series of polymerizations using TPEBIB as an initiator (Figure 2A and Table S1). The setup of the LED reactor is shown in Figure 3 and Figure S1. Monomer conversion at λ_max_ = 365 nm reached 31% after 6.5 h, however, with a broader dispersity = 2.18. A 99% monomer conversion with a product with a significantly lower dispersity = 1.13 was obtained at a lower energy (λ_max_ = 405 nm). However, a small higher-molecular-weight shoulder in the mass distribution was also observed (Figure 2A). Further increasing the wavelength toward the more visible light part of the spectrum resulted in 96% conversion with *Đ* ≤ 1.10 and a very monomodal mass distributions in 4–15 h under blue (λ_max_ = 470 nm), green (λ_max_ = 527 nm), or even red (λ_max_ = 630 nm) LED (Figure 2A). The blue LED (λ_max_ = 470 nm) was selected to perform further polymerizations owing to the faster reaction rate as well as maintaining excellent control over polymerization. This is in broad agreement with Barner-Kowollik and co-workers in that the absorption of the complex does not correlate with the most efficient reaction conditions and a red shift is observed from the maximum absorbance for the best reaction conditions (Figure 4).

Interestingly, even with irradiation at a lower energy, relative to any apparent significant absorption, we observe high monomer conversions over these prolonged reaction conditions with good, if not better, control over molecular weight and dispersity compared to a more intense higher energy UV light (Figure 4A). This is in broad agreement to that reported by Barner-Kowollik and co-workers with our study demonstrating that the molar extinction coefficient of the complex is not a reliable indication to select the optimum irradiation wavelength. We note that the free ligand does have a tail of the absorption band well into the visible region in a seemingly exponential decay and does not reach zero absorption (Figure 4B). Thus, this is consistent with our original observation that the important photon absorption is into the free ligand followed by energy transfer to the complexed ligand.^53^ We do note that a major difference between this data and the type reported by Barner-Kowollik *et al.* is that in their case, they take care to use identical amounts of photons, which could not be achieved with our commercial LED setup. Furthermore, we prioritized to take the polymerization reactions to high monomer conversion rather than time limit stopping reactions at low-to-moderate conversion. From the conversion plots as a function of irradiation wavelength, we identified the blue light-induced process (λ = 470 nm) as providing quite intriguing polymerization conditions given the minimal spectral overlap with the copper complex.

**Figure 5:**
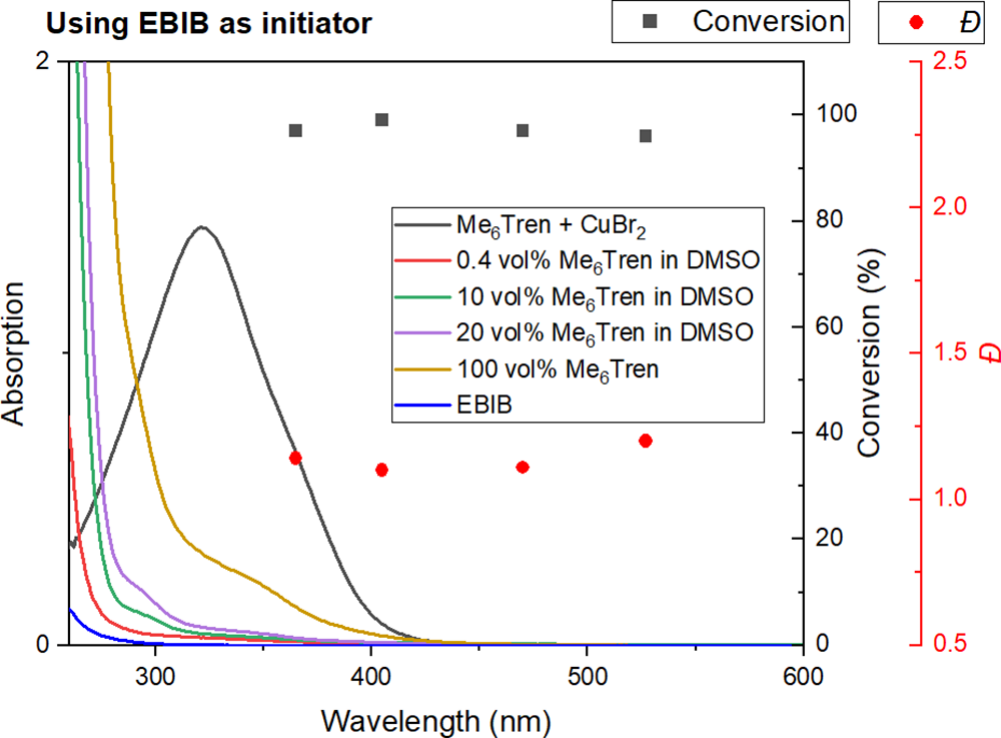
Wavelength-dependent polymerization of methyl acrylate obtained by LED irradiation with overlaid UV–Vis spectrum of CuBr_2_/Me_6_TREN in DMSO with reaction concentration, neat and diluted Me_6_TREN, and the EBIB initiator in DMSO. Black points (squares) showing % conversion and red points (circles) showing the polymer dispersity.

The molecular characteristics and in general the quality of the obtained polymers are highly dependent on the excited-state dynamics, the redox behavior, and the different species that can be generated upon UV irradiation. Since the AIE initiator has a significant absorption across the UV spectral range, it could thus potentially interfere with the photo-polymerization. The absorption spectrum of TPEBIB and spectral characterizations of different LEDs are shown in Figure 1. There is an overlap between the absorption spectrum of the initiator and the used UV LEDs (λ_max_ = 365 and 405 nm), indicating that the absorption by the initiator seems to be detrimental to the polymerization efficiency. To test this hypothesis, similar reactions were carried out using EBIB as the initiator, having very little to no absorption above 300 nm (Figure 5). As a result, polymers with low dispersity at high conversions were all accessible regardless of the chosen wavelength of the LED or the light intensity used, with a 94–99% monomer conversion and *Đ* = 1.08–1.24 depending on the exact conditions (Table S2). Thus, loss of control is observed when the initiator has more significant absorption of the incident light.

Subsequently, the effect of the intensity of the blue LED irradiation was further investigated. Lumidox II devices are calibrated with five discrete linearly stepped output stages. Stage 1 output has the least radiometric power, while stage 5 output is the highest. The details of the power and irradiance used are listed in Table S3. The kinetics of the polymerization of MA is demonstrated in Figure 2B, with five varying stages of applied intensity. The reactions were conducted using identical settings, with the temperature being 5 °C in the external cooling circulator. Despite the same targeted temperature, there is still a significant actual temperature difference of the reactions. Reaction temperatures were measured as 26, 40, 54, 66, and 90 °C, respectively, when the intensity was increased from stage 1/5 to 5/5. This results from the inability of the thermal deck attached to the lamp to not cool the reaction efficiently due to the intense radiation from the LED array. Nonetheless, good linear first-order kinetics throughout the reaction was observed for the two lowest intensities (stages 1/5 and 2/5). However, a further increase in the intensity resulted in a nonlinear relationship between ln[M]/[M_0_] and the reaction time, especially when the conversion of MA was >85%, suggesting the lack of control at a higher monomer conversion. Additionally, it is much more challenging to reach high conversions (>90%) if the reaction temperature is too high (Figure 2B, stage 5/5), attributed to increased termination occurring. This was further confirmed by the polymerization results conducted under the same light intensity but varying temperatures (Figure 2C). Considering both the good linear control and reasonable reaction rate, stage 2/5 was chosen for future polymerization reactions. The effect of catalyst concentration was also investigated (Figure S3) with the highest conversion (96%) and lowest *Đ* (1.09) achieved in 4 h with CuBr_2_:Me_6_Tren:initiator = 0.02:0.12:1.

**Figure 6:**
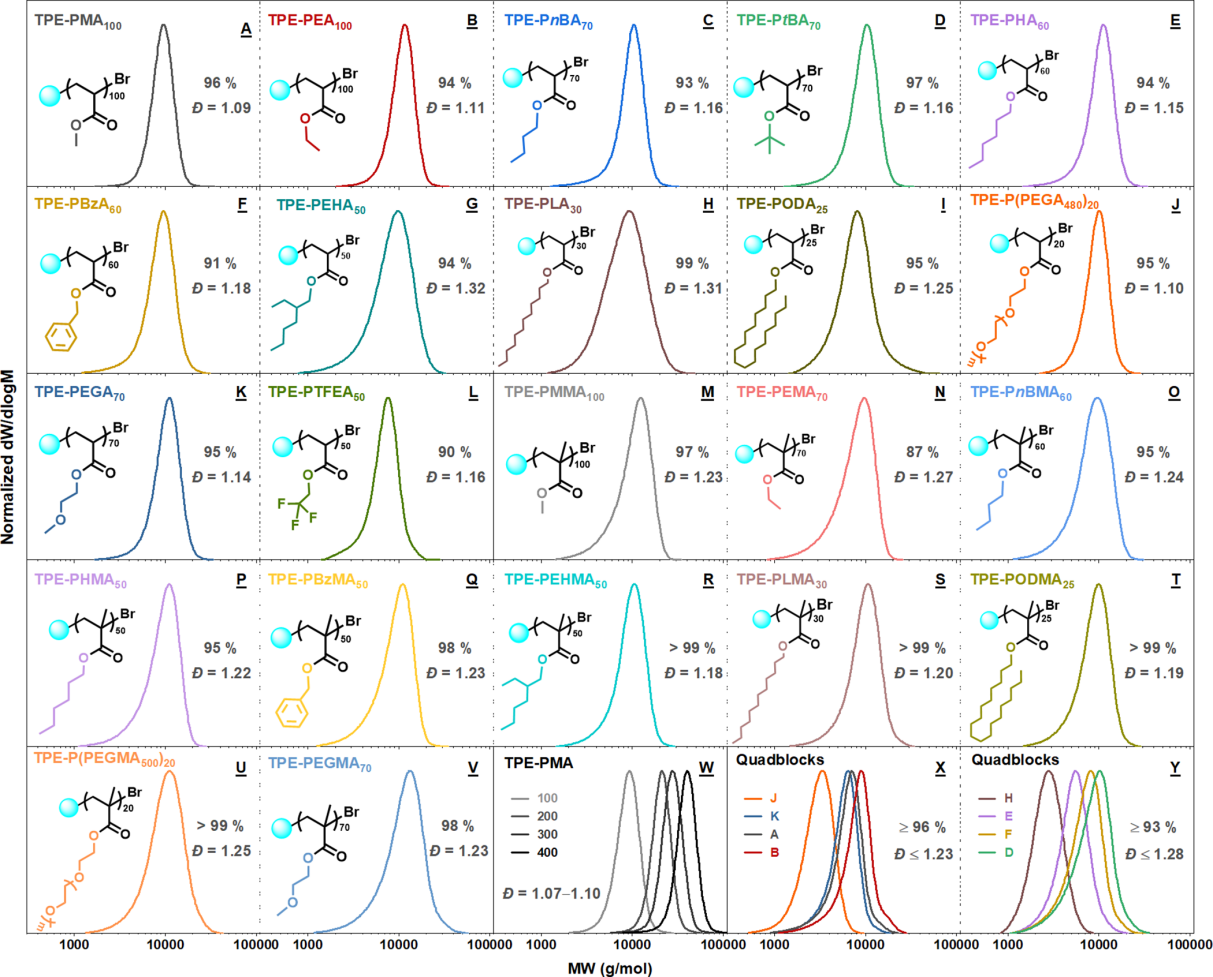
THF-SEC-derived molecular weight distributions of TPE-terminated (A–L) polyacrylates and (M–V) polymethacrylates with varying architectures using blue light-mediated Cu-RDRP. (W) THF-SEC of TPE-PMA targeting different DPs (100–400). (X, Y) THF-SEC of TPE-terminated tetrablock preparation by iterative monomer additions in one pot.

Using identical optimized conditions, a library of both well-defined hydrophilic and hydrophobic poly(meth)acrylates from >20 monomers was successfully synthesized with targeted *M*_n_ ≈ 9000 (Figure 6A–V and Table S4). These monomers include linear and branched (meth)acrylates with varying alkyl length (C1, C2, C4, C6, C8, C12, or C18). Additionally, fluorine-containing poly(trifluoroethyl acrylate) (PTFEA) was also successfully prepared (Figure 6L). In most cases, high conversions were reached (≥95%) with quite low dispersities ranging from 1.09 to 1.25. Significantly, the negligible deviation between the theoretical (*M*_n,th_) and experimental molecular weights (*M*_n,NMR_ and *M*_n,GPC_) (Table S4) as well as symmetrical monomodal SEC traces without tailing demonstrated excellent control.

Notably, the choice of solvent is crucial depending on the solubility of catalysts, TPEBIB initiator, and the resultant polymers and monomers. For the hydrophilic monomers, poly(ethylene glycol) methyl ether acrylate (PEGA_480_) and ethylene glycol methyl ether acrylate (EGA), and hydrophobic monomers with short alkyl chain, methyl acrylate and ethyl acrylate, DMSO was used as the solvent due to the good solubility of the resultant polymers, monomers, catalyst, and initiator. TPE-PMA_100_, TPE-PEA_100_, TPE-P(PEGA_480_)_20_, and TPE-PEGA_70_ with a narrow *Đ* ∼ 1.10 were obtained at a high monomer conversion (∼95%, 4–6 h) in DMSO (Figure 6A–B and J–K). However, with more hydrophobic polymers, for example, poly(*n*-butyl acrylate) (P*n*BA), employing DMSO as the solvent leads to less control (*Đ* = 1.53), resulting from polymerization-induced phase separation (PIPS) (Table S5). To perform polymerizations in a more controlled fashion, a series of solvents were compared with *n*BA as the monomer. DMF gave the best performance, giving polymers with monomodal distributions (*Đ* = 1.16) at a high monomer conversion (93%, 12 h) (Figure 6C). Other monomers, *tert*-butyl acrylate (*t*BA), hexyl acrylate (HA), and benzyl acrylate (BzA), were polymerized in DMF with well-defined polymers attained (*Đ* ∼ 1.16, conversion = 91–97%) (Figure 6C–F). Again, polymerization of monomers with longer alkyl chains (≥C8) in pure DMF were not well-controlled resulting from PIPS (Table S6). In an attempt to increase the solubility of polymers, THF was introduced as a cosolvent, giving a higher lauryl acrylate (LA) conversion (99%) than in dioxane (85%) with the similar *Đ* = 1.31. Also, poly(2-ethylhexyl acrylate) (PEHA) and poly(octadecyl acrylate) (ODA) were synthesized in DMF/THF mixtures (1/4) at high conversions (94 and 95%, respectively) with products having relatively low *Đ* (1.32 and 1.25, respectively) (Figure 6G–I).

Similarly, a series of polymethacrylates were prepared employing the same solvents used for polyacrylates with the same side chains. Initially, the identical conditions were used for polymerization of methyl methacrylate (MMA), furnishing a polymer with *Đ* = 1.39 at 83% monomer conversion (Table S7). To accelerate the reaction rate, the catalyst amount was tripled to CuBr_2_:Me_6_Tren:initiator = 0.06:0.36:1, which leads to a near full monomer conversion (98%) and a decreased *Đ* = 1.26. By replacing Me_6_Tren with *N*,*N*,*N*,*N*,*N*-pentamethyldiethylenetriamine (PMDETA), the dispersity was further narrowed to *Đ* = 1.23 (Figure 6M). Thus, CuBr_2_:PMDETA:initiator = 0.06:0.36:1 was adopted for the polymerization of various methacrylates. Dispersities decreased (from 1.27 to 1.19) without compromising near full conversions (>99%) within the same time frame (20 h) upon increasing the alkyl chain length of methacrylates, which evidenced the opposite trend of the case of acrylates (Figure 6A–V and Table S4).

We subsequently investigated the potential of this system in maintaining good control across a wide range of molar masses. Polymerizations of MA targeting degrees of polymerization (DP_n_) from 100 to 400 were performed, resulting in high monomer conversions within 4 h (90–96%), quite low *Đ* (1.07–1.10), and excellent agreement between theoretical and experimental molecular weights (Figure 6W and Table S8).

*In situ* chain extension is widely employed to both verify high end-group fidelity and allow easy access to multiblock functional copolymers with no need for purification between the iterative monomer additions. Chain extension from P(PEGA_480_) was attempted, employing identical previously developed conditions (Table S9). After 8 h, a near full monomer conversion (98%) was attained (*Đ* = 1.16) and a deoxygenated solution of a second block (EGA) in DMSO (1/1) and the catalyst were subsequently injected into the reaction mixture. In a similar manner, following several monomer additions, an amphiphilic tetrablock (TPE-P(PEGA_480_)_5_-*b*-PEGA_25_-*b*-PMA_15_-*b*-PEA_15_) was achieved (*Đ* = 1.23, M_n_ = 7000) (Figure 6X). Notably, chain extensions from the less active monomers were also attempted (Figure 6Y and Table S10). A tetrablock, TPE-PLA_8_-*b*-PHA_10_-*b*-PBzA_10_-*b*-P*t*BA_10_, was also prepared with the first two blocks reaching full conversions followed by 93% monomer conversions for the last two monomer additions. Although the dispersity tended to slightly increase upon the addition of the subsequent block, the obtained tetrablock still had a reasonable *Đ* = 1.28. Collectively, SEC analysis revealed monomodal peaks with a shift of the mass distribution to higher molecular weights upon the next block addition while maintaining low dispersities, thus suggesting good end-group fidelity at high monomer conversions.

Temporal control of this blue LED-mediated Cu-RDRP system was examined with the polymerization of MA in DMSO (Figure S4). An observable yet reproducible amount of polymer growth during the “off” cycles evidenced non-ideal temporal control, which agrees with other photo Cu-RDRP systems.^29,54,56^ The unexpected chain growth in the dark was attributed to the increased lifetime of residual activator after initial photoactivation.^57^ It was also reported that better temporal control could be achieved by decreasing the catalyst amount.^57^

**Figure 7:**
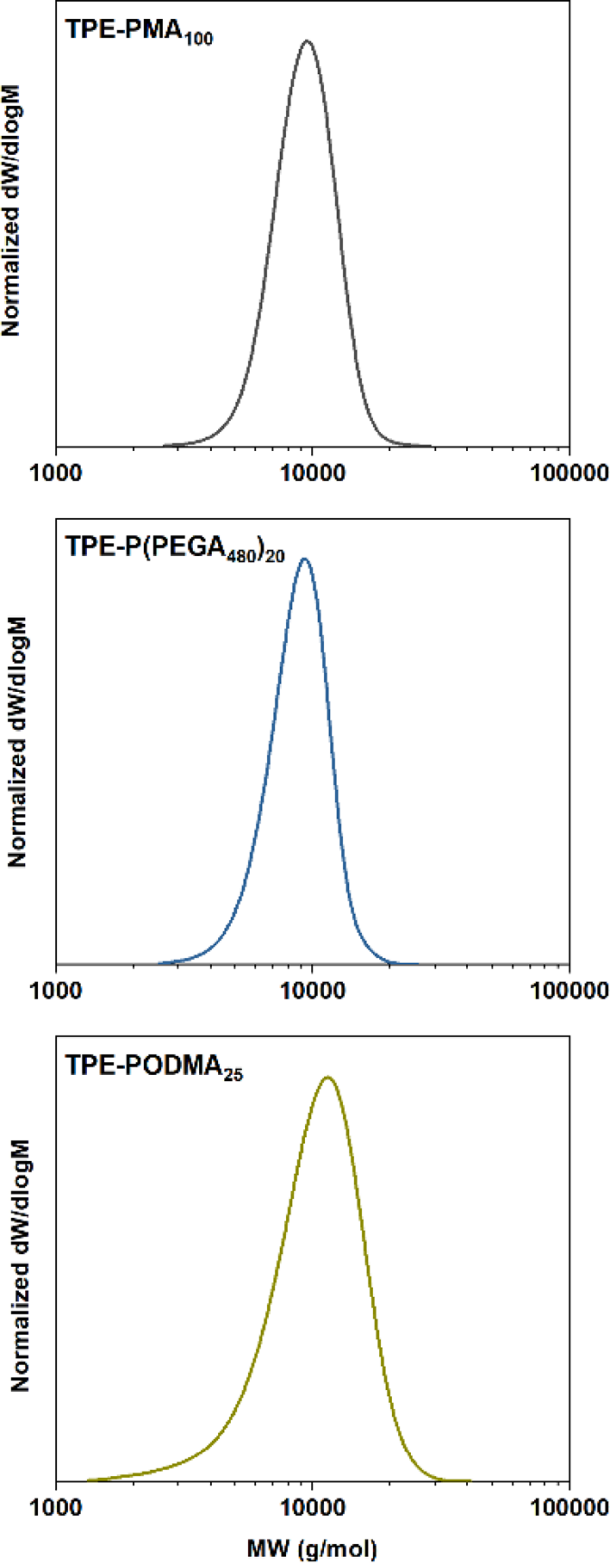
THF-SEC-derived molecular weight distribution of TPE-PMA_100_, TPE-P(PEGA_480_)_20_, and TPE-PODMA_25_, synthesized by blue light-mediated RDRP in a flow reactor.

There are advantages in using flow chemistry and especially when considering sustainability aspects of production. Thus, following our batch syntheses, we investigated this protocol using polymerization in a plug flow system using the same LED arrays in a commercially available flow reactor designed specifically for these arrays (Figure 3B and more details in Figure S2). The exposure volumes were set to 1.67 mL in total using 3.2 mm outer diameter (OD) with a 1.6 mm inner diameter (ID) tubing. MA, PEGA_480_, and ODMA were chosen for this comparison (Table 1). Competitive control and especially narrow dispersity were observed in the plug flow reactor under the same conditions (Figure 7). By comparing the results in batch and continuous flow reactions, polymers with similar molecular characteristics were obtained, with a slightly lower conversion in the case of flow reactions, which is attributed to the process implemented without stirring. All synthesized TPE-terminated polymers were characterized by NMR (Figures S5–S8). The TPE group incorporation into the polymers was exhibited by the signals δ = 7.15–6.77 ppm in ^1^H NMR spectrum (Figures S5 and S6) and δ = 150–120 ppm of ^13^C NMR spectrum (Figures S7 and S8). Additionally, the characteristic peaks were clearly assigned.

**Table 1:** Homopolymerizations of MA, PEGA_480_, and ODMA by Blue LED (470 nm)-Mediated Cu-RDRP Using TPEBIB in a Flow Reactor^a^

polymer^b^	residence time (h)	flow rate (μL/min)	Con. (%)^c^	*M*_n,th_ (g/mol)	*M*_n,SEC_ (g/mol)^d^	*Đ*
TPE-PMA_100_	4	6.96	92	8400	8800	1.08
TPE-P(PEGA_480_)_20_	6	4.64	91	9200	8300	1.08
TPE-PODMA_25_	20	1.39	84	7600	9000	1.21

a Flow reactor: light intensity: stage 2/5, 150 mW/well, 14.4 W in total, 0.12 W cm^–2^.

b Reaction conditions: for acrylates, [CuBr_2_]:[Me_6_Tren] = 0.02:0.12; for methacrylates, [CuBr_2_]:[PMDETA] = 0.06:0.36.

c Conversions were calculated according to ^1^H NMR in CDCl_3_.

d Determined by SEC employing THF as an eluent calibrated by narrow PMMA molecular weight standards.

**Figure 8:**
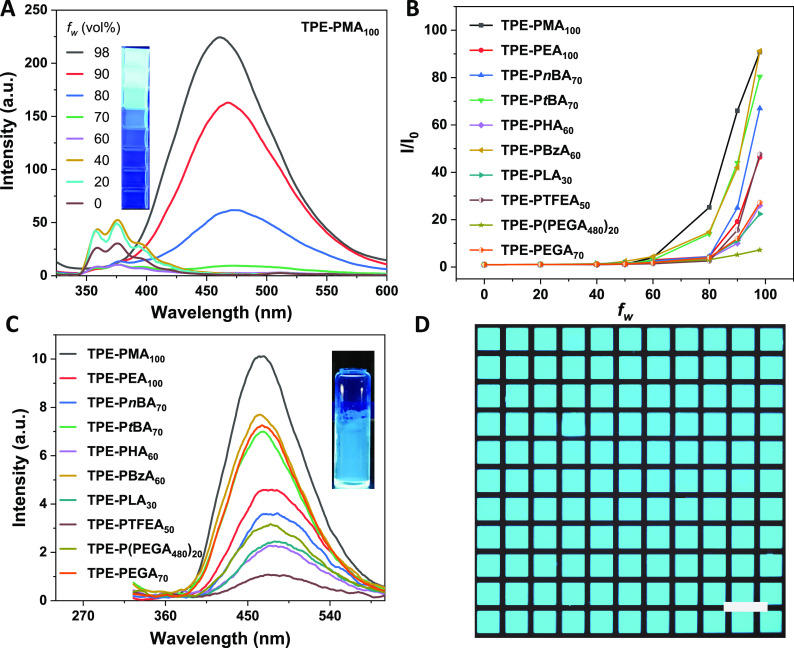
(A) PL spectra of TPE-PMA_100_ in water/THF mixtures with different water fractions (*f*_w_) measured at 20 °C. (B) Plots of *I/I*_0_ versus *f*_w_ for the TPE-terminated polymers (*I* is the PL intensity in the THF/water mixtures; *I*_0_ is the emission intensity in pure THF solution). (C) Emission of polymer films. (D) Two-dimensional fluorescent photopatterns of TPE-PMMA_100_. Scale bar = 200 μm. λ_ex_ = 310 nm for all the PL measurements with [Polymer] = 10 μM. The pictures in the insets were taken under a UV lamp.

Subsequently, some photophysical properties of the polymers were investigated to assess the functionality of the AIE-containing initiator post-polymerization. The AIE behaviors were examined in both THF/water mixtures and in the solid state (Figure 8 and Figure S9). All the polymers exhibited typical AIE properties due to the presence of the TPE moiety. For example, TPE-PMA_100_ emitted weakly when solubilized in pure THF. Upon gradual addition of water (a poor solvent for PMA) to the THF solution, the luminescence was slowly enhanced before the water fraction (*f*_w_) reached 80 vol %. Further increasing *f*_w_ to 98 vol % contributed to the strongly enhanced emission (Figure 8A). Notably, the photoluminescence intensity of TPE-PMA_100_ in 98% THF/water mixture is >90-fold higher than that in pure THF (*I*_0_) (Figure 8B). In a similar fashion, all other TPE-terminated polymers were demonstrated to be AIE-active. In addition, the solid state of these polymers was also measured to be emissive (Figure 8C). This is ascribed to the restriction of intramolecular motions of the TPE moiety in the aggregated state, which blocks the nonradiative decay pathway of the excited state to turn on the emission of the polymer.^58^

Given the efficient emission of the TPE-terminated polymers in both aggregated and solid states, their potential application in photopatterning was explored. A thin TPE-PMMA_100_ film was fabricated by spin-coating followed by exposure to UV light (λ = 330–380 nm) in air for 20 min at ambient temperature through a negative copper photomask. A two-dimensional fluorescent photopattern was readily generated (Figure 8D). The unexposed squares endured bright fluorescence, whereas the emission of the exposed regions was quenched, possibly resulting from the photooxidation process,^59^ giving a turn-off-type 2D photopattern. Significantly, the generation of such a fluorescent pattern by a photolithography technique demonstrated that these synthesized TPE-containing polymers are promising materials regarding optoelectronic applications.

## Conclusions

Thus, we have demonstrated the wavelength dependence of photoinduced Cu(II) reduction for controlled radical polymerization with simple commercially available LED systems. We find broad agreement with Barner-Kowollik *et al*. that the absorption of the copper(II) complex is not a reliable guide to reaction conditions using a commercial system designed to be utilized for routine synthesis. A red shift away from the absorption maximum is observed in accordance with the results achieved using a pulsed laser irradiation. Optimized conditions utilized visible light to give a library of AIE-active hydrophobic (meth)acrylates with side chains containing linear and branched alkyl groups as well as fluorine. It proved important to avoid absorption of the incident light by the initiator to achieve the best control of molecular weight and dispersity. The well-defined polymers and *in situ* generation of sequential multiblock copolymers show good targeted molecular weights, narrow chain length distributions, and high end-group fidelity at high monomer conversions, allowing for the extension of copper-mediated RDRP to give AIEgen-containing polymers. This strategy is compatible with both batch and plug flow reactors. Moreover, the TPE-terminated polymers exhibited excellent performance in photopatterning, enabling their potential applications in optoelectronics and other film- and bulk-based applications.

## Supporting Information Available

NMR spectra, GPC traces, PL spectra, experimental materials, instruments, and methods, setup pictures, detailed homopolymerization and multiblock copolymerization results, temporal control experiments, and additional data for photo-mediated Cu-RDRP of methyl acrylate, five stages’ output, homopolymerizations of a library of (meth)acrylates, effects of solvents, catalyst amount, and ligand to polymerization, synthesis of TPE-PMA, and *in situ* chain extension to prepare multiblock polymers (PDF)
