# Supplementary material for: Aggregation-Induced Emission Poly(meth)acrylates for Photopatterning via Wavelength-Dependent Visible-Light-Regulated Controlled Radical Polymerization in Batch and Flow Conditions
Source: Macromolecules. 2022 Nov 11;55(22):9908–17. doi: 10.1021/acs.macromol.2c01413 (PMC9686136; doi:10.1021/acs.macromol.2c01413)
Supplement: Supplementary file 1 — ma2c01413_si_001.pdf [file ma2c01413_si_001.pdf]

Supporting information

Aggregation induced emission poly(meth)acrylates for photopatterning *via* wavelength-dependent visible-light-regulated controlled radical polymerization in batch and flow conditions

Congkai Ma[†], *Ting Han*[‡], *Spyridon Efstathiou*[†], *Arkadios Marathianos*[†], *Hannes Houck*[†] *and David M. Haddleton*[*, †]

[†]Department of Chemistry, University of Warwick, Coventry, CV4 7AL, United Kingdom

[‡]Center for AIE Research, College of Materials Science and Engineering, Shenzhen University, Shenzhen 518060, China

Experimental Section

Materials

The monomers, methyl acrylate (MA, 99%), ethyl acrylate (EA, 99%), poly(ethylene glycol) methyl ether acrylate (average M_n 480) ($PEGA_{480}$), ethylene glycol methyl ether acrylate (EGA, 98%), *n*-butyl acrylate (*n*BA, ≥99%), *tert*-butyl acrylate (*t*BA, 98%), benzyl acrylate (BzA, ≥99%) and methyl methacrylate (MMA, 99%) were all purchased from Sigma-Aldrich and used as received and without removal of the inhibitor.

The other monomers, hexyl acrylate (HA, 98%), 2,2,2-trifluoroethyl acrylate (TFEA, 99%), lauryl acrylate (LA, 90%), 2-ethylhexyl acrylate (HEA. 98%), octadecyl acrylate (ODA, 97%), ethyl methacrylate (EMA, 99%), poly(ethylene glycol) methyl ether methacrylate (average M_n 500) ($PEGMA_{500}$), ethylene glycol methyl ether methacrylate (EGMA, 99%), *n*-butyl methacrylate (*n*BMA, 99%), *tert*-butyl methacrylate (*t*BMA, 98%), benzyl methacrylate (BzMA, 96%), hexyl methacrylate (HMA, 98%), 2,2,2-trifluoroethyl methacrylate (TFEMA, 99%), lauryl methacrylate (LMA, 96%), 2-ethylhexyl methacrylate (HEMA, 99%), and octadecyl methacrylate (ODMA, a mixture of stearyl and cetyl methacrylate) were all purchased from Sigma-Aldrich and used after removal of the inhibitor by running through a basic aluminium column.

Materials were purchased from Sigma-Aldrich or Fischer Scientific unless otherwise stated. Copper(II) bromide ($CuBr_2$, Sigma-Aldrich, 99 %), dimethyl sulfoxide (DMSO), tetrahydrofuran (THF), dimethylformamide (DMF), dioxane, isopropanol (IPA), toluene, and methanol (MeOH) were purchased from Sigma-Aldrich or Fischer Scientific and used as received.

Tris-(2-(dimethylamino)ethyl)amine (Me_6Tren) was synthesized according to a literature procedure[1] and stored in the fridge.

Instruments and Analysis

Nuclear Magnetic Resonance (NMR) spectra were recorded on Bruker DPX (300, 400 or 500 MHz) spectrometers in deuterated chloroform ($CDCl_3$) from Sigma-Aldrich. Monomer conversions were determined via ^1H NMR spectroscopy by comparing the integrals of monomeric vinyl protons to polymer side chain signals.

Size exclusion chromatography (SEC) measurements were conducted using an Agilent Infinity II MDS instrument equipped with differential refractive index (DRI), viscometry (VS), dual angle light scatter (LS) and multiple wavelength UV detectors. The system was equipped with 2 x PLgel Mixed C columns (300 x 7.5 mm) and a PLgel 5 µm guard column. The eluent is THF with 0.01 % BHT (butylated hydroxytoluene) additive. Samples were run at 1 mL/min at 30 °C. Poly(methyl methacrylate) and polystyrene standards (Agilent EasiVials) were used for calibration. Analyte samples were filtered through a GVHP membrane with 0.22 µm pore size before injection. Respectively, experimental molar mass ($M_{n,SEC}$) and dispersity ($Đ$) values of synthesized polymers were determined by conventional calibration using Agilent GPC/SEC software.

Fourier-transform infrared spectroscopy (FTIR) spectra were recorded on a Perkin Elmer Spectrum One FT-IR Golden gate ATR.

Electrospray Ionization Time-of-Flight (ESI-ToF) mass spectra were measured using either an Agilent 6130B

single Quad or a Bruker Compact, Bruker MaXis II, MaXis Plus and MaXis Impact Q-ToF.

Photoluminescence (PL) spectra were recorded on a Cary Eclipse Fluorescence Spectrophotometer from Agilent Technologies at 20 °C. The excitation and emission slits were both fixed to be 5 nm.

Lumidox II LED arrays were purchased from Analytical Sales. The 96-position arrays with diffuse mat and solid base were set at the powers specified. Irradiance (mW cm^{-2}) output of the arrays was measured at the surface of the diffuse mat using a ThorLabs S142C Integrating Sphere Photodiode Power Sensor with Silicon Detector, connected to a ThorLabs PM400 optical power meter at the emission wavelength as specified by the LED supplier.

Experimental procedures

Initiator TPEBIB synthesis

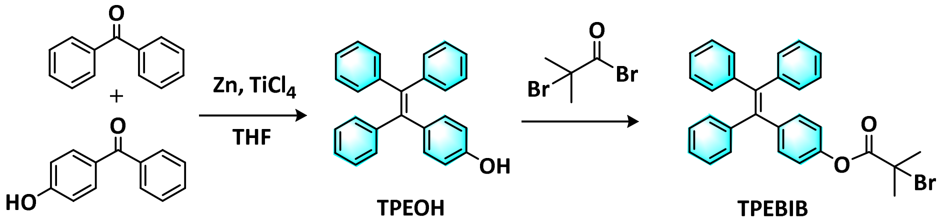

TPEOH was synthesized based on a literature procedure.[2] Benzophenone (1.82 g, 10 mmol), 4-hydroxybenzophenone (1.90 g, 10 mmol), zinc powder (1.60 g, 24 mmol) were dissolved in anhydrous THF. $TiCl_4$ (1.3 mL, 12 mmol) was injected dropwise into the mixture (cold) under nitrogen. After stirring for 0.5 h, the mixture was heated to reflux overnight. Dilute hydrochloric acid was added to quench the reaction after the reaction. The crude product was extracted with dichloromethane, washed with brine and then purified by silica-gel column using hexane/DCM as eluent. ^{1}H NMR (400 MHz, $CDCl_3$) δ 7.19 – 7.06 (m, 9H), 7.06 – 6.94 (m, 6H), 6.89 (d, J = 7.5 Hz, 2H), 6.56 (d, J = 7.6 Hz, 2H), 4.62 (s, 1H). TPEOH (0.5 g, 1.435 mmol), and triethylamine (0.25 ml, 1.722 mmol) were added to anhydrous THF (5ml) in a 25 mL three-necked bottle, which was cooled to 0 °C using an ice bath. Then a solution of 2-bromoisobutyryl bromide (BIBB, 0.27 ml, 2.152 mmol) was added dropwise. The suspension stirred at 0 °C for half an hour and was kept at room temperature for 2 days. The mixture was filtered, after which the solution was washed with saturated aqueous Na_2CO_3 and water. Rotary evaporation of the solution resulted in a yellow liquid, which was further purified by column chromatography using hexane/EA as eluent. TPEBIB: ^{1}H NMR ($CDCl_3$-*d*) δ 7.19 – 6.98 (m, 17H, ArH), 6.88 (d, *J* = 8.3 Hz, 2H, ArH), 2.03 (s, 6H, CH_3). ^{13}C NMR ($CDCl_3$-*d*) δ 170.25 (C), 149.23 (C), 143.68 (C), 143.59 (C), 143.53 (C), 141.82 (C), 141.59 (C), 139.97 (C), 132.52 (CH), 131.49 (CH), 131.44 (CH), 131.41 (CH), 127.96 (CH), 127.86 (CH), 127.80 (CH), 126.76 (CH), 126.71 (CH), 126.65 (CH), 120.37 (CH), 55.53 (C), 30.77 (CH_3). HRMS: *m/z* [M + Na]+ calcd. for $C_{29}H_{22}NaO$, 409.1563; found, 409.1567. FT-IR: v_{max} 3019, 2923, 1744, 1595, 1501, 1442, 1387, 1371, 1263, 1208, 1191, 1163, 1136, 1099, 1075, 1016, 877, 747, 700, 608 cm^{-1}.

Setup for photo reactions in batch

The setup for reactions in batch is illustrated in Figure S1. The blue LED, specifically Lumidox II 96-Position LED Arrays with diffuse Mat and Solid Base, was attached with a thermal transfer deck (used for controlling the temperature of the LED). The Para-dox Aluminum Reaction Block (8 mL) was put onto the top of the LED. The controller was attached to the LED to control the light intensity and exposure time. The thermal transfer deck was connected with a mini chiller using tubing to control the temperature. Beneath the deck, there is a hot plate to allow access for stirring. Before turning on the light, the whole setup was covered by a dark box.

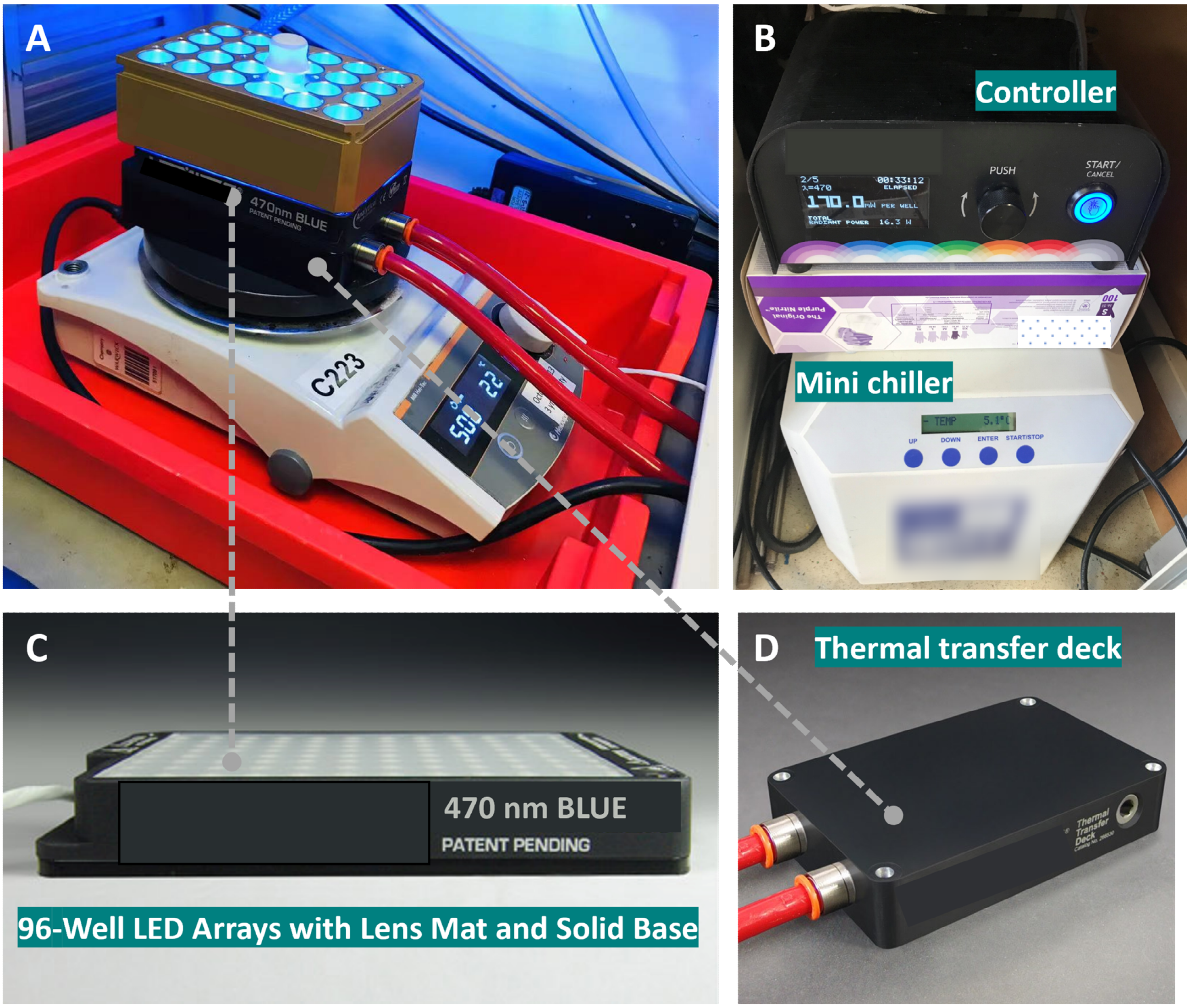

Figure S1 (A) The setup for photo reactions in batch using Lumidox II 96-Position LED Array; (B) Lumidox Gen II LED Controller and mini chiller; (C) The expended picture of the 96-Well Blue LED Array with Lens Mat and Solid Base; (D) The expended picture of thermal transfer deck.

Setup for photo reactions in flow

The setup for reactions in batch is illustrated in Figure S2. Similarly, the LED Array (with diffuse Mat and Solid Base) was attached with a thermal transfer deck. Instead using the block, the flow reactor with fitted tubing sits directly on top of the LED. Additionally, both the flow reactor and the thermal transfer deck were connected to the same mini chiller to control the reaction temperature. The degassed reactant mixture was injected to the tubes of the flow reactor using a gas-tight syringe by a Syringe pump. Before the light was turned on, the syringe, collection vial and the tubing outside of the reactor were all covered with foil. The whole volume of the flow reactor is 1.67 mL.

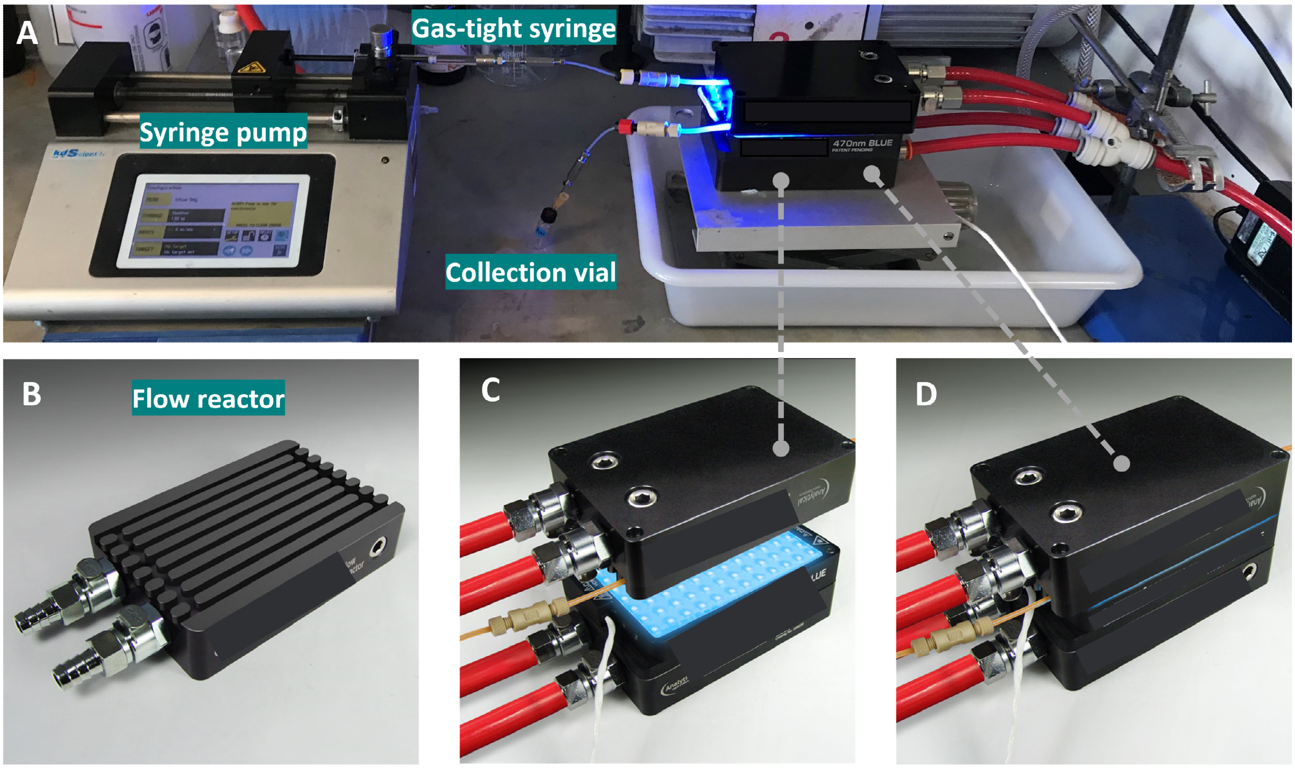

Figure S2 (A) The setup for photo reactions in flow using Lumidox II 96-Position LED Array; (B) The Para-Dox flow reactor; (C and D) The assembled flow reactor with LED and thermal transfer deck.

Procedure for photo Cu-RDRP of MA with targeted DP_n=100 in batch

The stock solution of Me_6TREN (17.8 µL), $CuBr_2$ (2.5 mg), and DMSO (5.0 mL) was prepared. Then the stock solution (0.5 mL, containing Me_6TREN : $CuBr_2$ = 0.12 equiv. : 0.02 equiv.), MA (0.5 mL, 100 equiv.), initiator TPEBIB (27.6 mg, 1 equiv.) and a stirring bar were added in a septum sealed vial. The mixture was subsequently deoxygenated by bubbling with nitrogen for 20 min, after which the vial was put into one of the wells of the Aluminum reaction block (**Figure S1**). Spontaneously, the mini chiller was set to be 5 °C. The LED was turned on from the controller to start the photo polymerization. Samples were taken periodically under a nitrogen blanket to determine the time needed for near quantitative conversion to be reached and passed through a short column of neutral alumina to remove dissolved copper salts prior to analysis by NMR and SEC. The polymer was purified by participating in cold water/methanol (1/1) mixture to remove catalyst and unreacted monomer.

Procedure for photo Cu-RDRP of EA with targeted DP_n=100 in batch

Similarly, the stock solution of Me_6TREN (14.7 µL), $CuBr_2$ (2.0 mg), and DMSO (5.0 mL) was prepared. Then the stock solution (0.5 mL, containing Me_6TREN : $CuBr_2$ = 0.12 equiv. : 0.02 equiv.), EA (0.5 mL, 100 equiv.), initiator TPEBIB (22.8 mg, 1 equiv.) and a stirring bar were added in a septum sealed vial. The mixture was subsequently deoxygenated by bubbling with nitrogen for 20 min, after which the vial was put into one of the wells of the metal holder on the top of the LED. Spontaneously, the mini chiller was set to be 5 °C. The LED was turned on from the controller to start the photo polymerization. Samples were taken periodically under a nitrogen blanket to determine the time needed for near quantitative conversion to be reached and passed through a short column of neutral alumina to remove dissolved copper salts prior to analysis by NMR and SEC. The polymer was purified by participating in cold water/methanol (1/1) mixture to remove catalyst and unreacted monomer.

Procedure for photo Cu-RDRP of nBA with targeted DP_n=70 in batch

Similarly, the stock solution of Me_6TREN (16.0 µL), $CuBr_2$ (2.2 mg), and DMF (5.0 mL) was prepared. Then the stock solution (0.5 mL, containing Me_6TREN : $CuBr_2$ = 0.12 equiv. : 0.02 equiv.), *n*BA (0.5 mL, 70 equiv.), initiator TPEBIB (24.8 mg, 1 equiv.) and a stirring bar were added in a septum sealed vial. The mixture was subsequently deoxygenated by bubbling with nitrogen for 20 min, after which the vial was put into one of the wells of the metal holder on the top of the LED. Spontaneously, the mini chiller was set to be 5 °C. The LED was turned on from the controller to start the photo polymerization. Samples were taken periodically under a nitrogen blanket to determine the time needed for near quantitative conversion to be reached and passed through a short column of neutral alumina to remove dissolved copper salts prior to analysis by NMR and SEC. The polymer was purified by participating in cold water/methanol (1/1) mixture to remove catalyst and unreacted monomer.

Procedure for photo Cu-RDRP of tBA with targeted DP_n=70 in batch

Similarly, the stock solution of Me_6TREN (15.8 µL), $CuBr_2$ (2.2 mg), and DMF (5.0 mL) was prepared. Then the stock solution (0.5 mL, containing Me_6TREN : $CuBr_2$ = 0.12 equiv. : 0.02 equiv.), *t*BA (0.5 mL, 70 equiv.), initiator TPEBIB (24.2 mg, 1 equiv.) and a stirring bar were added in a septum sealed vial. The mixture was subsequently

deoxygenated by bubbling with nitrogen for 20 min, after which the vial was put into one of the wells of the metal holder on the top of the LED. Spontaneously, the mini chiller was set to be 5 °C. The LED was turned on from the controller to start the photo polymerization. Samples were taken periodically under a nitrogen blanket to determine the time needed for near quantitative conversion to be reached and passed through a short column of neutral alumina to remove dissolved copper salts prior to analysis by NMR and SEC. The polymer was purified by participating in cold water/methanol (1/1) mixture to remove catalyst and unreacted monomer.

Procedure for photo Cu-RDRP of HA with targeted DP_n=60 in batch

Similarly, the stock solution of Me_6TREN (15.8 µL), $CuBr_2$ (2.2 mg), and DMF (5.0 mL) was prepared. Then the stock solution (0.5 mL, containing Me_6TREN : $CuBr_2$ = 0.12 equiv. : 0.02 equiv.), HA (0.52 mL, 60 equiv.), initiator TPEBIB (24.5 mg, 1 equiv.) and a stirring bar were added in a septum sealed vial. The mixture was subsequently deoxygenated by bubbling with nitrogen for 20 min, after which the vial was put into one of the wells of the metal holder on the top of the LED. Spontaneously, the mini chiller was set to be 5 °C. The LED was turned on from the controller to start the photo polymerization. Samples were taken periodically under a nitrogen blanket to determine the time needed for near quantitative conversion to be reached and passed through a short column of neutral alumina to remove dissolved copper salts prior to analysis by NMR and SEC. The polymer was purified by participating in cold water/methanol (1/2) mixture to remove catalyst and unreacted monomer.

Procedure for photo Cu-RDRP of BzA with targeted DP_n=60 in batch

Similarly, the stock solution of Me_6TREN (16.1 µL), $CuBr_2$ (2.2 mg), and DMF (5.0 mL) was prepared. Then the stock solution (0.5 mL, containing Me_6TREN : $CuBr_2$ = 0.12 equiv. : 0.02 equiv.), BzA (0.46 mL, 60 equiv.), initiator TPEBIB (24.9 mg, 1 equiv.) and a stirring bar were added in a septum sealed vial. The mixture was subsequently deoxygenated by bubbling with nitrogen for 20 min, after which the vial was put into one of the wells of the metal holder on the top of the LED. Spontaneously, the mini chiller was set to be 5 °C. The LED was turned on from the controller to start the photo polymerization. Samples were taken periodically under a nitrogen blanket to determine the time needed for near quantitative conversion to be reached and passed through a short column of neutral alumina to remove dissolved copper salts prior to analysis by NMR and SEC. The polymer was purified by participating in cold water/methanol (1/4) mixture to remove catalyst and unreacted monomer.

Procedure for photo Cu-RDRP of EHA with targeted DP_n=50 in batch

Similarly, the stock solution of Me_6TREN (16.0 µL), $CuBr_2$ (2.2 mg), and DMF (1.0 mL) was prepared. Then the stock solution (0.1 mL, containing Me_6TREN : $CuBr_2$ = 0.12 equiv. : 0.02 equiv.), EHA (0.52 mL, 50 equiv.), initiator TPEBIB (27.9 mg, 1 equiv.), THF (0.42 mL) and a stirring bar were added in a septum sealed vial. The mixture was subsequently deoxygenated by bubbling with nitrogen for 20 min, after which the vial was put into one of the wells of the metal holder on the top of the LED. Spontaneously, the mini chiller was set to be 5 °C. The LED was turned on from the controller to start the photo polymerization. Samples were taken periodically under a nitrogen blanket to determine the time needed for near quantitative conversion to be

reached and passed through a short column of neutral alumina to remove dissolved copper salts prior to analysis by NMR and SEC. The polymer was purified by participating in cold water/methanol (1/5) mixture and then dialysis in THF to remove catalyst and unreacted monomer.

Procedure for photo Cu-RDRP of LA with targeted DP_n=30 in batch

Similarly, the stock solution of Me_6TREN (20.4 µL), $CuBr_2$ (2.8 mg), and DMF (1.0 mL) was prepared. Then the stock solution (0.1 mL, containing Me_6TREN : $CuBr_2$ = 0.12 equiv. : 0.02 equiv.), LA (0.52 mL, 30 equiv.), initiator TPEBIB (24.9 mg, 1 equiv.), THF (0.42 mL) and a stirring bar were added in a septum sealed vial. The mixture was subsequently deoxygenated by bubbling with nitrogen for 20 min, after which the vial was put into one of the wells of the metal holder on the top of the LED. Spontaneously, the mini chiller was set to be 5 °C. The LED was turned on from the controller to start the photo polymerization. Samples were taken periodically under a nitrogen blanket to determine the time needed for near quantitative conversion to be reached and passed through a short column of neutral alumina to remove dissolved copper salts prior to analysis by NMR and SEC. The polymer was purified by participating in cold water/methanol (1/10) mixture to remove catalyst and unreacted monomer.

Procedure for photo Cu-RDRP of TFEA with targeted DP_n=50 in batch

Similarly, the stock solution of Me_6TREN (20.2 µL), $CuBr_2$ (2.8 mg), and DMF (2.0 mL) was prepared. Then the stock solution (0.2 mL, containing Me_6TREN : $CuBr_2$ = 0.12 equiv. : 0.02 equiv.), TFEA (0.4 mL, 50 equiv.), initiator TPEBIB (31.4 mg, 1 equiv.), THF (0.3 mL) and a stirring bar were added in a septum sealed vial. The mixture was subsequently deoxygenated by bubbling with nitrogen for 20 min, after which the vial was put into one of the wells of the metal holder on the top of the LED. Spontaneously, the mini chiller was set to be 5 °C. The LED was turned on from the controller to start the photo polymerization. Samples were taken periodically under a nitrogen blanket to determine the time needed for near quantitative conversion to be reached and passed through a short column of neutral alumina to remove dissolved copper salts prior to analysis by NMR and SEC. The polymer was purified by participating in cold water/methanol (1/3) mixture to remove catalyst and unreacted monomer.

Procedure for photo Cu-RDRP of ODA with targeted DP_n=25 in batch

Similarly, the stock solution of Me_6TREN (15.8 µL), $CuBr_2$ (2.2 mg), and DMF (1.0 mL) was prepared. Then the stock solution (0.1 mL, containing Me_6TREN : $CuBr_2$ = 0.12 equiv. : 0.02 equiv.), ODA (400 mg, 30 equiv.), initiator TPEBIB (24.9 mg, 1 equiv.), THF (0.5 mL) and a stirring bar were added in a septum sealed vial. The mixture was subsequently deoxygenated by bubbling with nitrogen for 20 min, after which the vial was put into one of the wells of the metal holder on the top of the LED. Spontaneously, the mini chiller was set to be 5 °C. The LED was turned on from the controller to start the photo polymerization. Samples were taken periodically under a nitrogen blanket to determine the time needed for near quantitative conversion to be reached and passed through a short column of neutral alumina to remove dissolved copper salts prior to analysis by NMR and SEC. The polymer was purified by participating in cold methanol mixture and then dialysis in THF to remove catalyst and unreacted monomer.

Procedure for photo Cu-RDRP of $PEGA_{480}$ with targeted DP_n=20 in batch

Similarly, the stock solution of Me_6TREN (14.6 µL), $CuBr_2$ (2.0 mg), and DMSO (5.0 mL) was prepared. Then the stock solution (0.5 mL, containing Me_6TREN : $CuBr_2$ = 0.12 equiv. : 0.02 equiv.), $PEGA_{480}$ (436 mg, 100 equiv.), initiator TPEBIB (22.6 mg, 1 equiv.) and a stirring bar were added in a septum sealed vial. The mixture was subsequently deoxygenated by bubbling with nitrogen for 20 min, after which the vial was put into one of the wells of the metal holder on the top of the LED. Spontaneously, the mini chiller was set to be 5 °C. The LED was turned on from the controller to start the photo polymerization. Samples were taken periodically under a nitrogen blanket to determine the time needed for near quantitative conversion to be reached and passed through a short column of neutral alumina to remove dissolved copper salts prior to analysis by NMR and SEC. The polymer was purified by participating in cold diethyl ether to remove catalyst and unreacted monomer.

Procedure for photo Cu-RDRP of EGA with targeted DP_n=70 in batch

Similarly, the stock solution of Me_6TREN (16.0 µL), $CuBr_2$ (2.2 mg), and DMSO (5.0 mL) was prepared. Then the stock solution (0.5 mL, containing Me_6TREN : $CuBr_2$ = 0.12 equiv. : 0.02 equiv.), EGA (0.45 mL, 70 equiv.), initiator TPEBIB (24.9 mg, 1 equiv.) and a stirring bar were added in a septum sealed vial. The mixture was subsequently deoxygenated by bubbling with nitrogen for 20 min, after which the vial was put into one of the wells of the metal holder on the top of the LED. Spontaneously, the mini chiller was set to be 5 °C. The LED was turned on from the controller to start the photo polymerization. Samples were taken periodically under a nitrogen blanket to determine the time needed for near quantitative conversion to be reached and passed through a short column of neutral alumina to remove dissolved copper salts prior to analysis by NMR and SEC. The polymer was purified by participating in cold diethyl ether to remove catalyst and unreacted monomer.

Procedure for photo Cu-RDRP of MMA with targeted DP_n=100 in batch

The stock solution of PMDETA (35.1 µL), $CuBr_2$ (6.3 mg), and DMSO (5.0 mL) was prepared. Then the stock solution (0.5 mL, containing PMDETA : $CuBr_2$ = 0.36 equiv. : 0.06 equiv.), MMA (0.5 mL, 100 equiv.), initiator TPEBIB (23.2 mg, 1 equiv.) and a stirring bar were added in a septum sealed vial. The mixture was subsequently deoxygenated by bubbling with nitrogen for 20 min, after which the vial was put into one of the wells of the metal holder (**Figure S1**). Spontaneously, the mini chiller was set to be 5 °C. The LED was turned on from the controller to start the photo polymerization. Samples were taken periodically under a nitrogen blanket to determine the time needed for near quantitative conversion to be reached and passed through a short column of neutral alumina to remove dissolved copper salts prior to analysis by NMR and SEC. The polymer was purified by participating in cold water/methanol (1/1) mixture to remove catalyst and unreacted monomer.

Procedure for photo Cu-RDRP of EMA with targeted DP_n=70 in batch

Similarly, the stock solution of PMDETA (34.5 µL), $CuBr_2$ (6.2 mg), and DMF (5.0 mL) was prepared. Then the stock solution (0.5 mL, containing PMDETA : $CuBr_2$ = 0.36 equiv. : 0.06 equiv.), EMA (0.4 mL, 70 equiv.), initiator TPEBIB (22.8 mg, 1 equiv.) and a stirring bar were added in a septum sealed vial. The mixture was subsequently deoxygenated by bubbling with nitrogen for 20 min, after which the vial was put into one of the wells of the metal holder on the top of the LED. Spontaneously, the mini chiller was set to be 5 °C. The LED was turned on from the controller to start the photo polymerization. Samples were taken periodically under a nitrogen blanket to determine the time needed for near quantitative conversion to be reached and passed through a

short column of neutral alumina to remove dissolved copper salts prior to analysis by NMR and SEC. The polymer was purified by participating in cold water/methanol (1/1) mixture to remove catalyst and unreacted monomer.

Procedure for photo Cu-RDRP of nBMA with targeted DP_n=60 in batch

Similarly, the stock solution of PMDETA (34.6 µL), $CuBr_2$ (6.2 mg), and DMF (5.0 mL) was prepared. Then the stock solution (0.5 mL, containing PMDETA : $CuBr_2$ = 0.36 equiv. : 0.06 equiv.), *n*BMA (0.44 mL, 60 equiv.), initiator TPEBIB (23.0 mg, 1 equiv.) and a stirring bar were added in a septum sealed vial. The mixture was subsequently deoxygenated by bubbling with nitrogen for 20 min, after which the vial was put into one of the wells of the metal holder on the top of the LED. Spontaneously, the mini chiller was set to be 5 °C. The LED was turned on from the controller to start the photo polymerization. Samples were taken periodically under a nitrogen blanket to determine the time needed for near quantitative conversion to be reached and passed through a short column of neutral alumina to remove dissolved copper salts prior to analysis by NMR and SEC. The polymer was purified by participating in cold water/methanol (1/1) mixture to remove catalyst and unreacted monomer.

Procedure for photo Cu-RDRP of HMA with targeted DP_n=50 in batch

Similarly, the stock solution of PMDETA (34.3 µL), $CuBr_2$ (6.1 mg), and DMF (5.0 mL) was prepared. Then the stock solution (0.5 mL, containing PMDETA : $CuBr_2$ = 0.36 equiv. : 0.06 equiv.), HMA (0.45 mL, 50 equiv.), initiator TPEBIB (22.7 mg, 1 equiv.) and a stirring bar were added in a septum sealed vial. The mixture was subsequently deoxygenated by bubbling with nitrogen for 20 min, after which the vial was put into one of the wells of the metal holder on the top of the LED. Spontaneously, the mini chiller was set to be 5 °C. The LED was turned on from the controller to start the photo polymerization. Samples were taken periodically under a nitrogen blanket to determine the time needed for near quantitative conversion to be reached and passed through a short column of neutral alumina to remove dissolved copper salts prior to analysis by NMR and SEC. The polymer was purified by participating in cold water/methanol (1/2) mixture to remove catalyst and unreacted monomer.

Procedure for photo Cu-RDRP of BzMA with targeted DP_n=50 in batch

Similarly, the stock solution of PMDETA (38.2 µL), $CuBr_2$ (6.8 mg), and DMF (5.0 mL) was prepared. Then the stock solution (0.5 mL, containing PMDETA : $CuBr_2$ = 0.36 equiv. : 0.06 equiv.), BzMA (0.43 mL, 50 equiv.), initiator TPEBIB (25.2 mg, 1 equiv.) and a stirring bar were added in a septum sealed vial. The mixture was subsequently deoxygenated by bubbling with nitrogen for 20 min, after which the vial was put into one of the wells of the metal holder on the top of the LED. Spontaneously, the mini chiller was set to be 5 °C. The LED was turned on from the controller to start the photo polymerization. Samples were taken periodically under a nitrogen blanket to determine the time needed for near quantitative conversion to be reached and passed through a short column of neutral alumina to remove dissolved copper salts prior to analysis by NMR and SEC. The polymer was purified by participating in cold water/methanol (1/4) mixture to remove catalyst and unreacted monomer.

Procedure for photo Cu-RDRP of EHMA with targeted DP_n=50 in batch

Similarly, the stock solution of PMDETA (42.3 µL), $CuBr_2$ (7.5 mg), and DMF (1.0 mL) was prepared. Then the stock solution (0.1 mL, containing PMDETA : $CuBr_2$ = 0.36 equiv. : 0.06 equiv.), EHMA (0.63 mL, 50 equiv.), initiator TPEBIB (28.0 mg, 1 equiv.), THF (0.53 mL) and a stirring bar were added in a septum sealed vial. The mixture was subsequently deoxygenated by bubbling with nitrogen for 20 min, after which the vial was put into one of the wells of the metal holder on the top of the LED. Spontaneously, the mini chiller was set to be 5 °C. The LED was turned on from the controller to start the photo polymerization. Samples were taken periodically under a nitrogen blanket to determine the time needed for near quantitative conversion to be reached and passed through a short column of neutral alumina to remove dissolved copper salts prior to analysis by NMR and SEC. The polymer was purified by participating in cold water/methanol (1/5) mixture and then dialysis in THF to remove catalyst and unreacted monomer.

Procedure for photo Cu-RDRP of LMA with targeted DP_n=30 in batch

Similarly, the stock solution of PMDETA (35.0 µL), $CuBr_2$ (6.2 mg), and DMF (2.0 mL) was prepared. Then the stock solution (0.2 mL, containing PMDETA : $CuBr_2$ = 0.36 equiv. : 0.06 equiv.), LMA (0.41 mL, 30 equiv.), initiator TPEBIB (23.2 mg, 1 equiv.), THF (0.3 mL) and a stirring bar were added in a septum sealed vial. The mixture was subsequently deoxygenated by bubbling with nitrogen for 20 min, after which the vial was put into one of the wells of the metal holder on the top of the LED. Spontaneously, the mini chiller was set to be 5 °C. The LED was turned on from the controller to start the photo polymerization. Samples were taken periodically under a nitrogen blanket to determine the time needed for near quantitative conversion to be reached and passed through a short column of neutral alumina to remove dissolved copper salts prior to analysis by NMR and SEC. The polymer was purified by participating in cold water/methanol (1/10) mixture to remove catalyst and unreacted monomer.

Procedure for photo Cu-RDRP of ODMA with targeted DP_n=25 in batch

Similarly, the stock solution of PMDETA (38.4 µL), $CuBr_2$ (6.8 mg), and DMF (1.0 mL) was prepared. Then the stock solution (0.1 mL, containing PMDETA : $CuBr_2$ = 0.36 equiv. : 0.06 equiv.), ODMA (432 mg, 25 equiv.), initiator TPEBIB (25.4 mg, 1 equiv.), THF (0.5 mL) and a stirring bar were added in a septum sealed vial. The mixture was subsequently deoxygenated by bubbling with nitrogen for 20 min, after which the vial was put into one of the wells of the metal holder on the top of the LED. Spontaneously, the mini chiller was set to be 5 °C. The LED was turned on from the controller to start the photo polymerization. Samples were taken periodically under a nitrogen blanket to determine the time needed for near quantitative conversion to be reached and passed through a short column of neutral alumina to remove dissolved copper salts prior to analysis by NMR and SEC. The polymer was purified by participating in cold methanol mixture and then dialysis in THF to remove catalyst and unreacted monomer.

Procedure for photo Cu-RDRP of $PEGMA_{500}$ with targeted DP_n=20 in batch

Similarly, the stock solution of PMDETA (33.8 µL), $CuBr_2$ (6.0 mg), and DMSO (5.0 mL) was prepared. Then the stock solution (0.5 mL, containing PMDETA : $CuBr_2$ = 0.36 equiv. : 0.06 equiv.), $PEGMA_{480}$ (450 mg, 100 equiv.), initiator TPEBIB (22.4 mg, 1 equiv.) and a stirring bar were added in a septum sealed vial. The mixture was

subsequently deoxygenated by bubbling with nitrogen for 20 min, after which the vial was put into one of the wells of the metal holder on the top of the LED. Spontaneously, the mini chiller was set to be 5 °C. The LED was turned on from the controller to start the photo polymerization. Samples were taken periodically under a nitrogen blanket to determine the time needed for near quantitative conversion to be reached and passed through a short column of neutral alumina to remove dissolved copper salts prior to analysis by NMR and SEC. The polymer was purified by participating in cold diethyl ether to remove catalyst and unreacted monomer.

Procedure for photo Cu-RDRP of EGMA with targeted DP_n=70 in batch

Similarly, the stock solution of PMDETA (33.5 µL), $CuBr_2$ (6.0 mg), and DMSO (5.0 mL) was prepared. Then the stock solution (0.5 mL, containing PMDETA : $CuBr_2$ = 0.36 equiv. : 0.06 equiv.), EGMA (0.45 mL, 70 equiv.), initiator TPEBIB (22.2 mg, 1 equiv.) and a stirring bar were added in a septum sealed vial. The mixture was subsequently deoxygenated by bubbling with nitrogen for 20 min, after which the vial was put into one of the wells of the metal holder on the top of the LED. Spontaneously, the mini chiller was set to be 5 °C. The LED was turned on from the controller to start the photo polymerization. Samples were taken periodically under a nitrogen blanket to determine the time needed for near quantitative conversion to be reached and passed through a short column of neutral alumina to remove dissolved copper salts prior to analysis by NMR and SEC. The polymer was purified by participating in cold diethyl ether to remove catalyst and unreacted monomer.

Procedure for photo Cu-RDRP of MA with targeted DP_n=100 in flow

The stock solution of Me_6TREN (16.0 µL), $CuBr_2$ (2.2 mg), and DMSO (4.5 mL) was prepared. Then the stock solution (0.3 mL, containing Me_6TREN : $CuBr_2$ = 0.12 equiv. : 0.02 equiv.), MA (0.3 mL, 100 equiv.), and initiator TPEBIB (16.6 mg, 1 equiv.) were added in a septum sealed vial. The mixture was subsequently sonicated and then deoxygenated by bubbling with nitrogen for 20 min, after which a gas-tight syringe was used to transfer the reactant (**Figure S2**). The flow rate of the syringe pump was set to be 6.96 µL/min to allow a 4 h radiation time. Spontaneously, the mini chiller was set to be 10 °C. The measured temperature of the reaction is 30 °C. The LED was turned on from the controller to start the photo polymerization. Upon all the reactants were injected, the inlet of the photo reactor was switched to 2.1 mL degassed solvent employing the same flow rate. Samples were taken and passed through a short column of neutral alumina to remove dissolved copper salts prior to analysis by NMR and SEC.

Procedure for photo Cu-RDRP of $PEGA_{480}$ with targeted DP_n=20 in flow

The stock solution of Me_6TREN (10.9 µL), $CuBr_2$ (1.5 mg), and DMSO (4.0 mL) was prepared. Then the stock solution (0.4 mL, containing Me_6TREN : $CuBr_2$ = 0.12 equiv. : 0.02 equiv.), $PEGA_{480}$ (0.3 mL, 20 equiv.), and initiator TPEBIB (16.9 mg, 1 equiv.) were added in a septum sealed vial. The mixture was subsequently sonicated and then deoxygenated by bubbling with nitrogen for 20 min, after which a gas-tight syringe was used to transfer the reactant (**Figure S2**). The flow rate of the syringe pump was set to be 4.64 µL/min to allow a 6 h radiation time. Spontaneously, the mini chiller was set to be 10 °C. The measured temperature of the reaction is 30 °C. The LED was turned on from the controller to start the photo polymerization. Upon all the reactants were injected, the inlet of the photo reactor was switched to 2.1 mL degassed solvent employing the same flow rate. Samples were taken and passed through a short column of neutral alumina to remove

dissolved copper salts prior to analysis by NMR and SEC.

Procedure for photo Cu-RDRP of ODMA$_{480}$ with targeted DP$_n$=25 in flow

The stock solution of PMDETA (22.2 µL), $CuBr_2$ (3.9 mg), and DMF (1.0 mL) was prepared. Then the stock solution (0.1 mL, containing PMDETA : $CuBr_2$ = 0.36 equiv. : 0.06 equiv.), ODMA (250 mg, 25 equiv.), initiator TPEBIB (14.7 mg, 1 equiv.), and THF (0.3 mL) were added in a septum sealed vial. The mixture was subsequently sonicated and then deoxygenated by bubbling with nitrogen for 20 min, after which a gas-tight syringe was used to transfer the reactant (**Figure S2**). The flow rate of the syringe pump was set to be 1.39 µL/min to allow a 20 h radiation time. Spontaneously, the mini chiller was set to be 10 °C. The measured temperature of the reaction is 30 °C. The LED was turned on from the controller to start the photo polymerization. Upon all the reactants were injected, the inlet of the photo reactor was switched to 2.1 mL degassed solvent employing the same flow rate. Samples were taken and passed through a short column of neutral alumina to remove dissolved copper salts prior to analysis by NMR and SEC.

Photopatterning

The fluorescent photopatterns were taken on a fluorescence optical microscope (Olympus IX73) under a UV light source (330–380 nm). The photopatterns were generated by UV irradiating the polymer thin films through a photomask for 20 min in air at room temperature. The photomask used herein was a pattern, in which the square areas were opaque and coated with copper whereas grid the grid lines were transparent glass substrate. The photo-irradiation process was conducted using UV light from a Changtuo CHF-XM500M Mercury Lamp with a distance of 25 cm. The incident light intensity was about 18.5 mW/cm^{-2}.

Table S1 Photo mediated Cu-RDRP of methyl acrylate in batch using UV nail box or LED with varying wavelengths initiated by TPEBIB.

Photo method	***Intensity***	***Time (h)***	***Conversion (%)***	***$M_{n,th}$ (g/mol)***	***$M_{n,SEC}$ (g/mol)***	***Đ***
UV Box (360 nm)	–	20	41	4000	4300	1.43
UV LED (365 nm)	45mW/well	20	14	1700	1700	1.60
UV LED (365 nm)	195mW/well	6.5	31	3200	2400	2.18
**UV LED (405 nm)**	**50mW/well**	**6.5**	**84**	7700	**9700**	**1.09**
**UV LED (405 nm)**	**110mW/well**	**3**	**92**	8400	**10500**	**1.18**
**UV LED (405 nm)**	**175mW/well**	**2**	**99**	9000	**10200**	**1.13**
**Blue LED (470 nm)**	**170mW/well**	**4**	**96**	8800	**8300**	**1.09**
**Green LED (527 nm)**	**180mW/well**	**6.5**	**96**	8800	**11400**	**1.07**

Table S2 Photo mediated Cu-RDRP of methyl acrylate in batch using UV nail box or LED with varying wavelengths initiated by EBIB.

Photo method	***Intensity***	***Time (h)***	***Conversion (%)***	***$M_{n,th}$ (g/mol)***	***$M_{n,SEC}$ (g/mol)***	***Đ***
UV Box (360 nm)	–	2	99	8800	8800	1.14
UV LED (365 nm)	195mW/well	2	97	8600	8100	1.24
**UV LED (405 nm)**	**50mW/well**	**2**	**94**	**8400**	**8600**	**1.08**
**UV LED (405 nm)**	**110mW/well**	**2**	**98**	**8800**	**10200**	**1.09**
**UV LED (405 nm)**	**175mW/well**	**2**	**99**	**8800**	**7700**	**1.10**
**Blue LED (470 nm)**	**170mW/well**	**4**	**97**	8600	**6800**	**1.11**
**Green LED (527 nm)**	**180mW/well**	**6.5**	**96**	8900	**5200**	**1.20**

Table S3 Five stages output (radiometric power) of Lumidox II 96-Well LED Arrays with Lens Mat and Solid Base with their corresponding power per well, total power and irradiance.

Stage	***Intensity***					
	**UV 365 nm**			**UV 405 nm**		
1/5	45 mW/Well	4.3 W	40 mW cm^{-2}	50 mW/Well	4.8 W	48 mW cm^{-2}
2/5	95 mW/Well	9.1 W	80 mW cm^{-2}	110 mW/Well	10.6 W	0.10 W cm^{-2}
3/5	150 mW/Well	14.4 W	0.12 W cm^{-2}	175 mW/Well	16.8 W	0.16 W cm^{-2}
4/5	195 mW/Well	18.7 W	0.16 W cm^{-2}	240 mW/Well	23.0 W	0.21 W cm^{-2}
5/5	295 mW/Well	28.3 W	0.24 W cm^{-2}	365 mW/Well	35.0 W	0.31 W cm^{-2}
Stage	***Intensity***					
	**Blue 470 nm**			**Green 527 nm**		
1/5	80 mW/Well	7.7 W	60 mW cm^{-2}	45 mW/Well	4.3 W	35 mW cm^{-2}
2/5	150 mW/Well	14.4 W	0.12 W cm^{-2}	85 mW/Well	8.2 W	60 mW cm^{-2}
3/5	220 mW/Well	21.1 W	0.18 W cm^{-2}	115 mW/Well	11.0 W	85 mW cm^{-2}
4/5	280 mW/Well	26.9 W	0.23 W cm^{-2}	145 mW/Well	13.9 W	0.10 W cm^{-2}
5/5	400 mW/Well	38.4 W	0.32 W cm^{-2}	180 mW/Well	17.3 W	0.13 W cm^{-2}
Stage	***Intensity***					
	**Red 630 nm**					
1/5	55 mW/Well	5.2 W	40 mW cm^{-2}			
2/5	105 mW/Well	10.0 W	80 mW cm^{-2}			
3/5	155 mW/Well	14.0 W	0.12 W cm^{-2}			
4/5	210 mW/Well	20.1 W	0.16 W cm^{-2}			
5/5	305 mW/Well	29.2 W	0.24 W cm^{-2}			

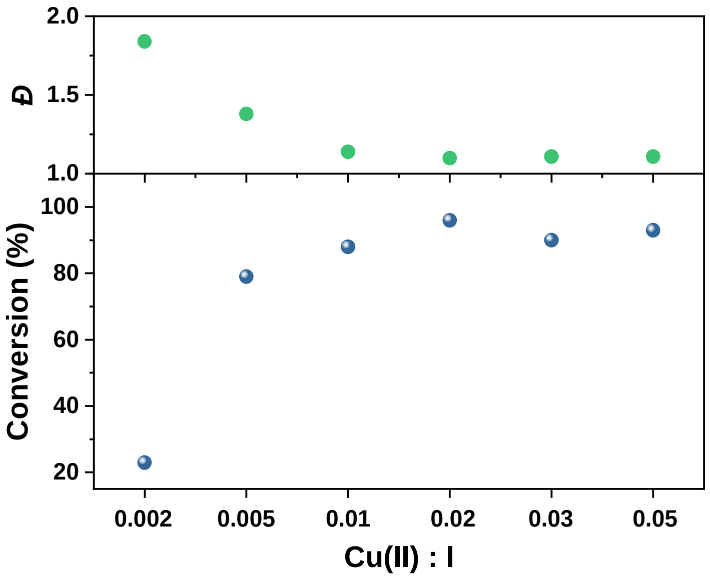

Figure S3 The effects on monomer conversion and polymer dispersity of catalyst concentrations. I is initiator.

Table S4 Homopolymerizations of a library of (meth)acrylates by blue-LED mediated Cu-RDRP using TPEBIB in batch

Polymers [a]	***Time (h)***	***Con.*** [b] ***(%)***	***$M_{n,th}$*** [b] ***(g/mol)***	***$M_{n,SEC}$*** [c] ***(g/mol)***	***$M_{n,NMR}$*** [d] ***(g/mol)***	***Đ***	***Solvents***
**TPE-PMA$_{100}$**	4	96	8670	8700	8900	1.09	DMSO
**TPE-PEA$_{100}$**	6	94	9900	10200	8000	1.11	DMSO
**TPE-P*n*BA$_{70}$**	12	93	8800	8600	8400	1.16	DMF
**TPE-P*t*BA$_{70}$**	10	97	9200	8600	8600	1.16	DMF
**TPE-PHA$_{60}$**	10	94	9300	9300	9600	1.15	DMF
**TPE-PBzA$_{60}$**	12	91	9400	7800	8800	1.18	DMF
**TPE-PEHA$_{50}$**	20	94	9200	7000	10400	1.32	DMF / THF
**TPE-PLA$_{30}$**	20	99	7700	7200	7700	1.31	DMF / THF
**TPE-PODA$_{25}$**	40	95	8200	7000	9900	1.25	DMF / THF
**TPE-P(PEGA$_{480}$)$_{20}$**	6	95	9620	8800	10100	1.10	DMSO
**TPE-PEGA$_{70}$**	4	95	9200	9400	9900	1.14	DMSO
**TPE-PTFEA$_{50}$**	20	90	7400	6400	6000	1.16	DMF / THF
**TPE-PMMA$_{100}$**	18	97	10200	9100	9000	1.23	DMSO
**TPE-PEMA$_{70}$**	20	87	7400	6600	6700	1.27	DMF
**TPE-P*n*BMA$_{60}$**	20	95	8600	7400	8600	1.24	DMF
**TPE-PHMA$_{50}$**	20	95	8600	8000	9000	1.22	DMF
**TPE-PBzMA$_{50}$**	20	98	9100	7900	9100	1.23	DMF
**TPE-PEHMA$_{50}$**	20	100	10500	8500	11800	1.18	DMF / THF
**TPE-PLMA$_{30}$**	20	100	8100	8600	9400	1.20	DMF / THF
**TPE-PODMA$_{25}$**	20	100	9000	7900	8600	1.19	DMF / THF
**TPE-P(PEGMA$_{500}$)$_{20}$**	10	100	10500	8800	14000	1.25	DMSO
**TPE-PEGMA$_{70}$**	10	95	10100	9500	13200	1.32	DMSO

[a] Reaction conditions: For acrylates, [$CuBr_2$]: [Me_6Tren] = 0.02 : 0.12; For methacrylates, [$CuBr_2$]: [PMDETA] = 0.06 : 0.36. [b] Conversions were calculated according to ^{1}H NMR in $CDCl_3$. [c] Determined by SEC employing THF as eluent calibrated by narrow PMMA molecular weight standards. [d] Determined by comparing the integrals of aromatic protons of the initiator to polymer side chain signals.

Table S5 The effects of solvents to polymerization involved *n*-butyl acrylate (*n*BA) as a model monomer

Solvent	***Time (h)***	***Con.(%)***	$M_{n,th}$ ***(g/mol)***	$M_{n,SEC}$ ***(g/mol)***	***Đ***
DMSO	8	99	9400	11500	1.53
IPA	20	84	8000	8300	1.22
Dioxane/MeOH	20	87	8300	9900	1.22
Toluene/MeOH	20	97	9200	20900	3.81
DMF	12	93	8800	8600	1.16

Table S6 The effects of solvents to polymerization involved lauryl acrylate (LA) as a model monomer

Solvent	***Time (h)***	***Con.(%)***	$M_{n,th}$ ***(g/mol)***	$M_{n,SEC}$ ***(g/mol)***	***Đ***
DMF	12	97	9800	9200	2.59
DMF/Dioxane	18	85	8400	8300	1.31
DMF/THF	20	99	7600	7200	1.31

Table S7 The effects of catalyst amount and ligand to polymerization involved methacrylate (MMA) as a model monomer

Polymer	***Time (h)***	***Catalyst***	***Con.(%)***	$M_{n,SEC}$ ***(g/mol)***	***Đ***
**TPE-PMMA$_{100}$**	21	Cu(II) : Me_6Tren = 0.02 : 0.12	83	7800	1.39
	21	Cu(II) : Me_6Tren = 0.06 : 0.36	98	8800	1.26
	18	Cu(II) : PMDETA = 0.06 : 0.36	97	9100	1.23

Table S8 The synthesis of TPE-PMA targeting varying DP=100–400 using photo Cu-RDRP

DP	***Time (h)***	***Con.(%)***	$M_{n,th}$ ***(g/mol)***	$M_{n,SEC}$ ***(g/mol)***	***Đ***
100	4	96	8800	8300	1.10
200	4	92	17700	19800	1.07
300	4	92	24100	26000	1.08
400	4	90	31300	37400	1.08

Table S9 The in situ chain extension from $P(PEGA_{480})_5$ to prepare multiblock polymers initiated by TPEBIB using photo Cu-RDRP

Blocks	***Time (h)***	***Con.(%)***	***$M_{n,th}$ (g/mol)***	***$M_{n,SEC}$ (g/mol)***	***Đ***
$P(PEGA_{480})_5$	b1-8 h	98	2800	2700	1.16
EGA_{25}	b2-10 h	96	6000	5200	1.16
MA_{15}	b3-8 h	97	7400	5600	1.20
EA_{15}	b4-13 h	98	8800	7000	1.23

Table S10 The in situ chain extension from PLA_8 to prepare multiblock polymers initiated by TPEBIB using photo Cu-RDRP

Blocks	***Time (h)***	***Con.(%)***	***$M_{n,th}$ (g/mol)***	***$M_{n,SEC}$ (g/mol)***	***Đ***
LA_8	b1-18 h	100	2400	2500	1.14
HA_{10}	b2-24 h	100	4000	4800	1.18
BzA_{10}	b3-24 h	93	5500	6200	1.23
tBA_{10}	b4-24 h	93	6700	7300	1.28

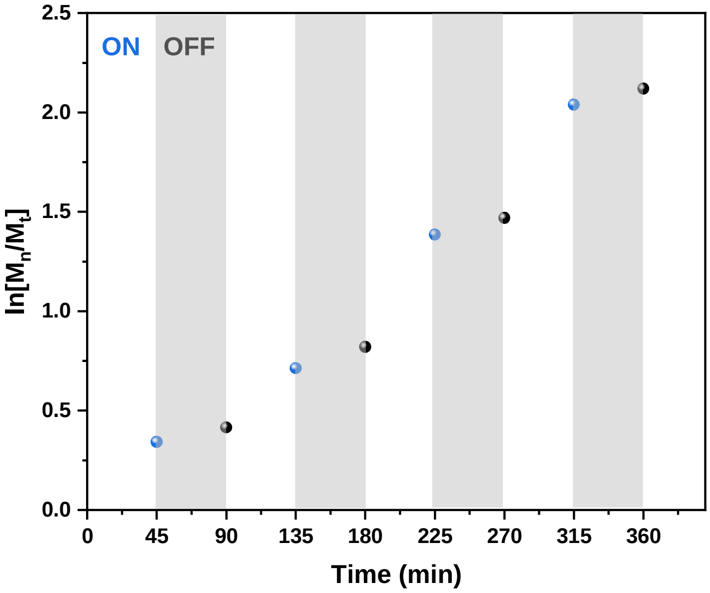

Figure S4 Temporal control experiments for the blue-light Cu-RDRP of MA in DMSO initiated by TPEBIB.

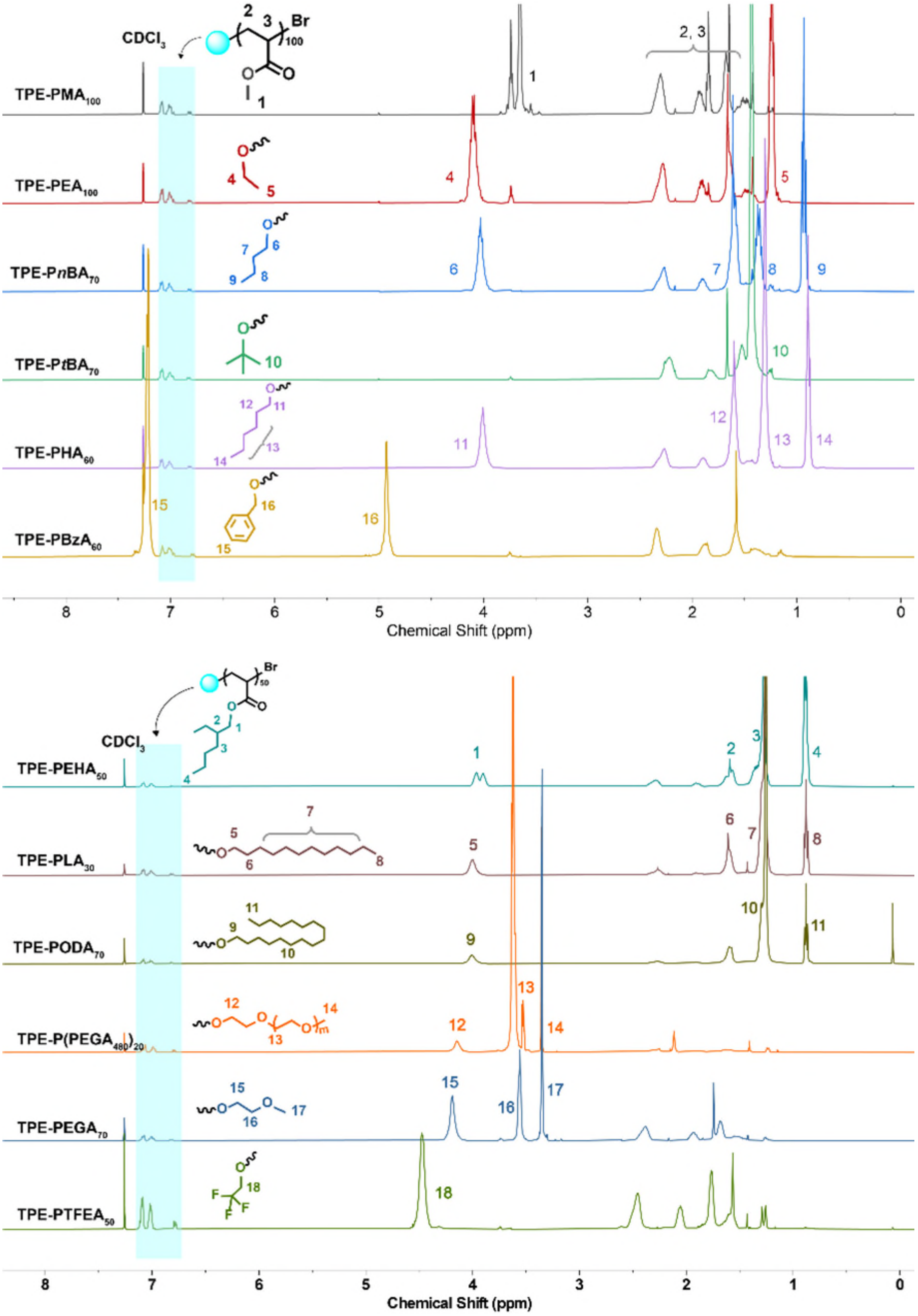

Figure S5 ^{1}H NMR of synthesized TPE-terminated polyacrylates.

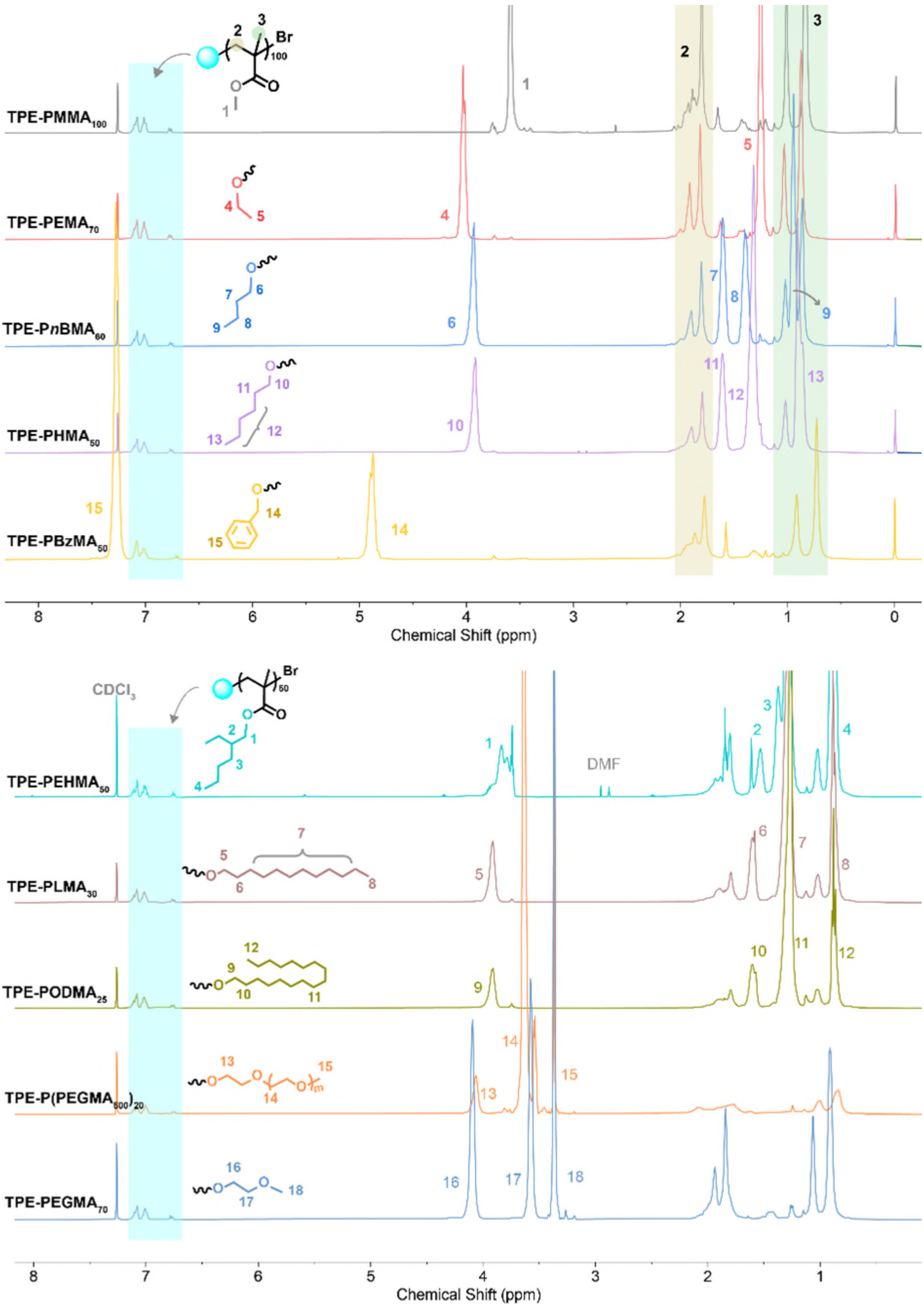

Figure S6 ^{1}H NMR of synthesized TPE-terminated polymethacrylates.

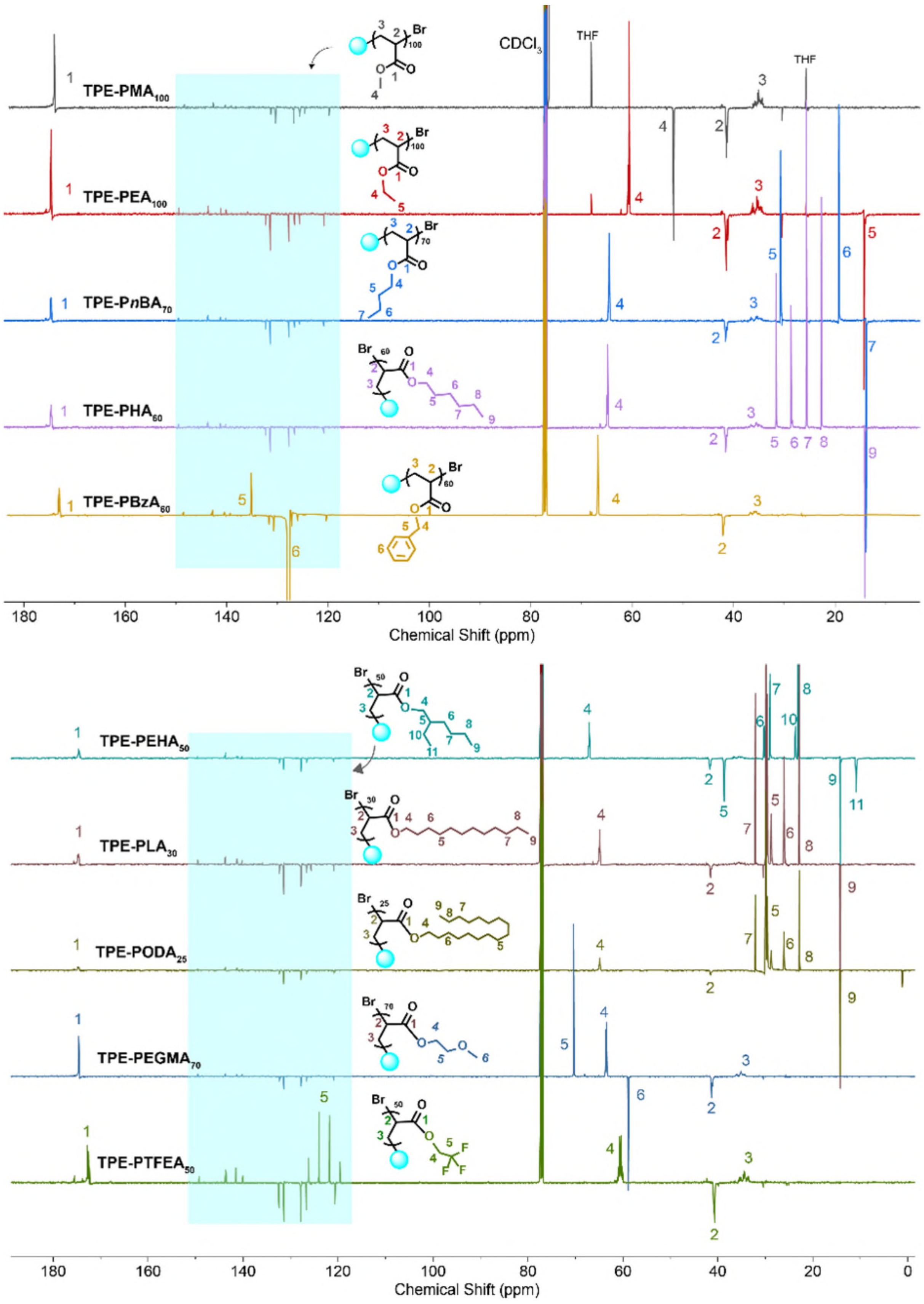

Figure S7 ^{13}C NMR of synthesized TPE-terminated polyacrylates.

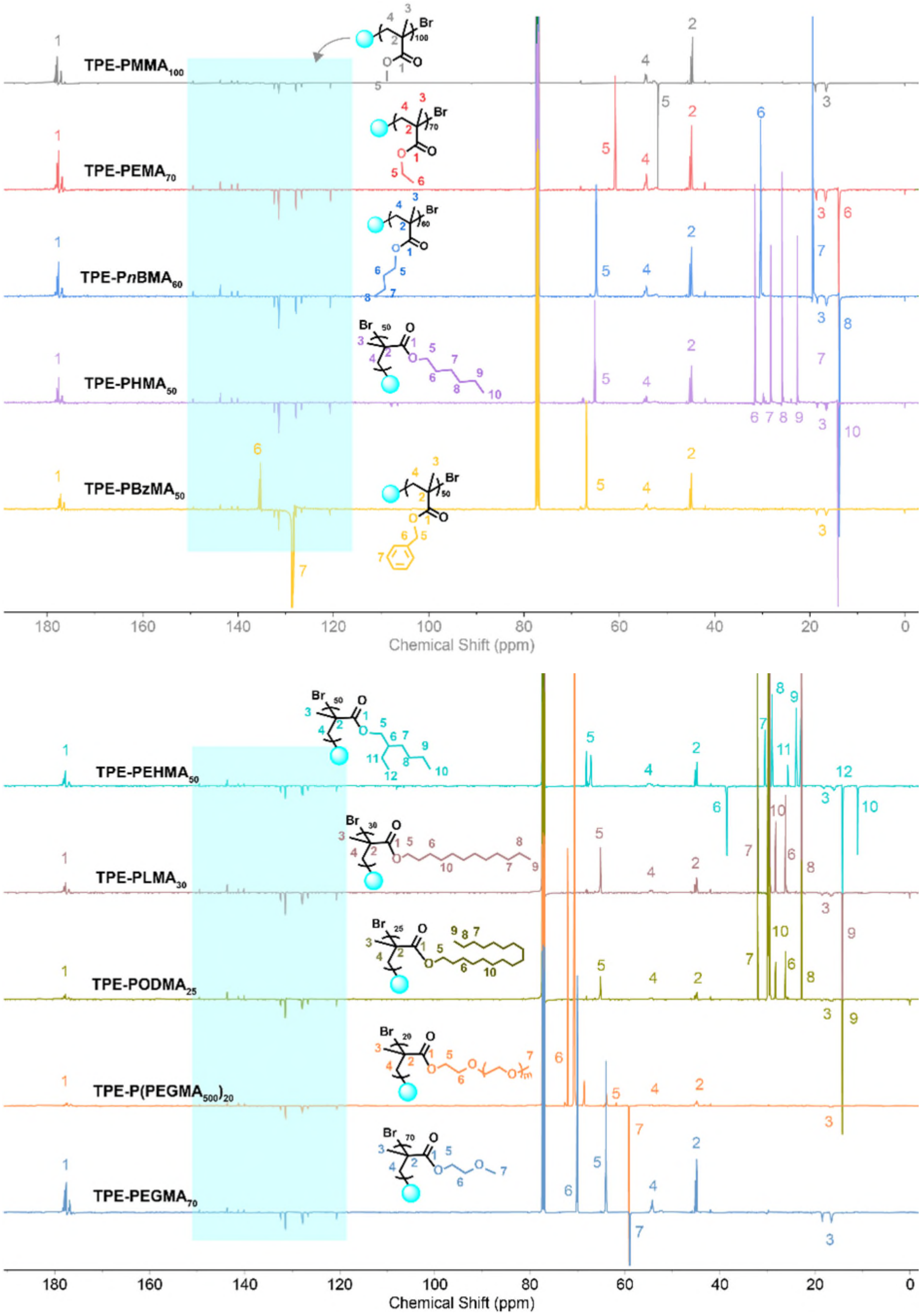

Figure S8 ^{13}C NMR of synthesized TPE-terminated polymethacrylates.

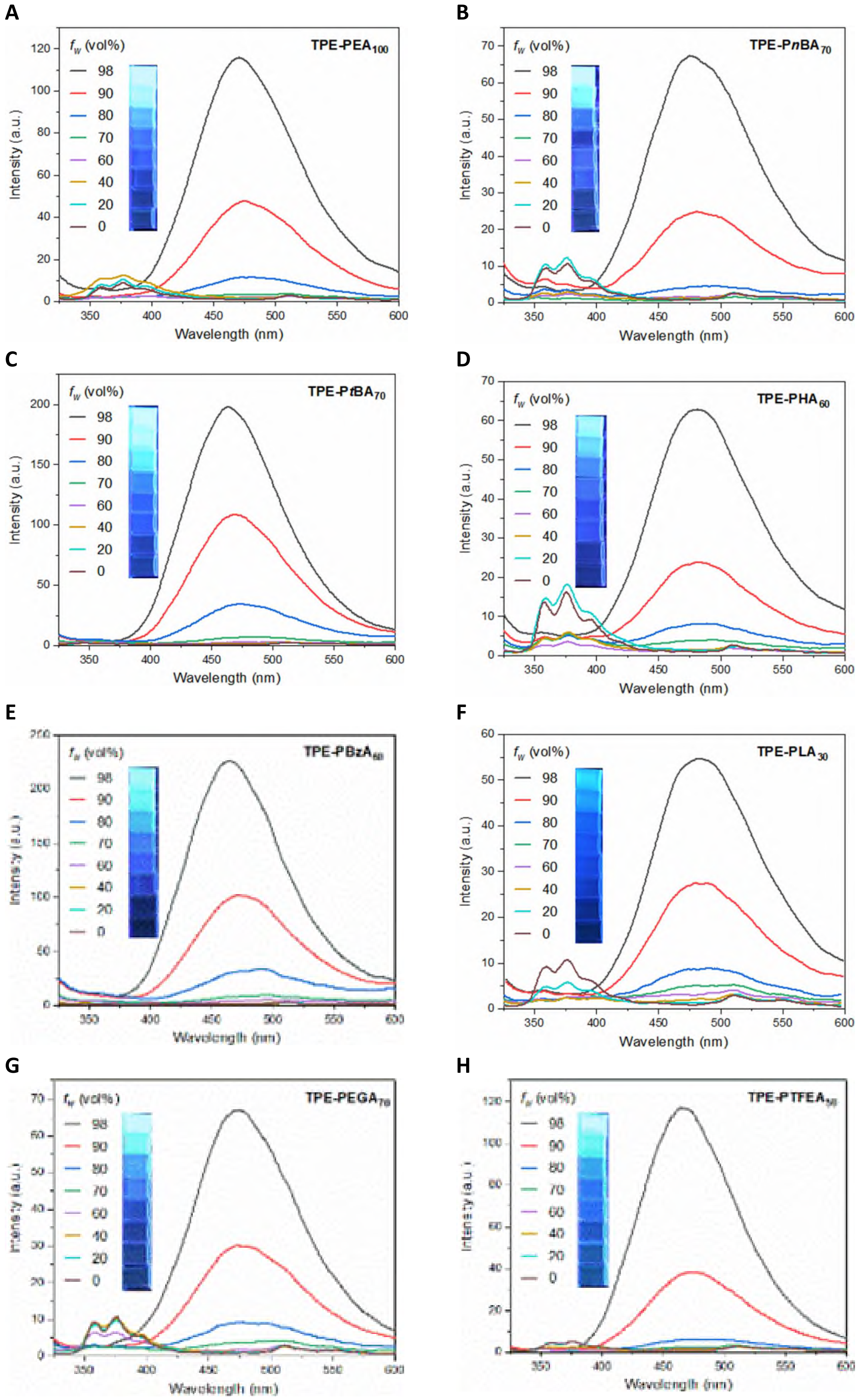

Figure S9 The PL spectra of different homopolymers in THF/water mixtures with different water fractions (f_w) measured at 20 °C with [polymer] = 10 μM. The inserted pictures were taken under a UV lamp (λ = 302 nm).

Reference

(1) Ciampolini, M.; Nardi, N., Five-Coordinated High-Spin Complexes of Bivalent Cobalt, Nickel, andCopper with Tris(2-dimethylaminoethyl)amine. *Inorganic Chemistry* **1966,** *5*, 41-44.

(2) Zhao, Y.; Kwok, R. T.; Lam, J. W.; Tang, B. Z., A highly fluorescent AIE-active theranostic agent with anti-tumor activity to specific cancer cells. *Nanoscale* **2016,** *8*, 12520-12523.
